# Safety Considerations for Natural Products with Adaptogenic and Immunomodulating Activities

**DOI:** 10.3390/ph18081208

**Published:** 2025-08-15

**Authors:** Chen Jia Wen Liang, Herman J. Woerdenbag, Corine Ekhart, Annabella Vitalone, Florence P. A. M. van Hunsel

**Affiliations:** 1 Pharmacy Master Programme, School of Science and Engineering, University of Groningen, Antonius Deusinglaan 1, 9713 AV Groningen, The Netherlands; c.j.w.liang@student.rug.nl; 2 Department of Pharmaceutical Technology and Biopharmacy, Groningen Research Institute of Pharmacy (GRIP), University of Groningen, Antonius Deusinglaan 1, 9713 AV Groningen, The Netherlands; h.j.woerdenbag@rug.nl; 3 Netherlands Pharmacovigilance Centre Lareb, Goudsbloemvallei 7, 5237 MH ’s-Hertogenbosch, The Netherlands; c.ekhart@lareb.nl; 4 Department of Physiology and Pharmacology ‘Vittorio Erspamer’, Sapienza University of Rome, Piazzale Aldo Moro 5, 00185 Rome, Italy; annabella.vitalone@uniroma1.it; 5 Department of PharmacoTherapy, -Epidemiology & -Economics, Groningen Research Institute of Pharmacy (GRIP), University of Groningen, Antonius Deusinglaan 1, 9713 AV Groningen, The Netherlands

**Keywords:** adaptogens, animal products, drug-related adverse reactions, herb–drug interactions, herbal drugs, immunomodulators, individual case safety reports (ICSRs), fungi, natural products, phytovigilance, VigiBase

## Abstract

**Background/Objectives**: Natural products with claimed adaptogenic and/or immunomodulatory effects are widely used in traditional medicine systems across Eurasia. These include herbal remedies (e.g., *Panax ginseng*), fungi (e.g., *Ganoderma lucidum*), and animal-derived substances (e.g., propolis from *Apis mellifera*). Despite their popularity, the safety profiles of these products—particularly concerning adverse events (AEs) and serious adverse events (SAEs)—remain insufficiently understood. This study aimed to assess the safety profiles of adaptogenic and immunomodulatory natural products through a scoping review of published human studies and an analysis of individual case safety reports (ICSRs) from the WHO-UMC VigiBase database. **Methods**: A scoping review was conducted using PubMed (1980–2024) in line with PRISMA-ScR guidelines. Eligible studies included randomized and non-randomized clinical trials and case reports in humans focused on safety outcomes. Data extraction followed the Joanna Briggs Institute (JBI) standardized template. ICSRs from VigiBase were analyzed by product type, AE type and seriousness, and demographic characteristics. The data were further organized to highlight the 15 most frequently reported products and their top five System Organ Classes (SOCs) and Preferred Terms (PTs). **Results**: The scoping review identified 51 natural products with reported adaptogenic and/or immunomodulatory properties. This included 285 clinical trials and 119 case studies on single-ingredient products and 54 clinical trials and 21 case studies on multi-ingredient preparations. Common AEs included gastrointestinal, dermatological, hepatic, cardiovascular, and immunological reactions. SAEs were rare but reported for *Echinacea purpurea*, *Silybum marianum*, and *Camellia sinensis.* From Vigibase, 45,042 ICSRs were retrieved for 49 natural products: 10,702 for single-ingredient and 34,340 for multi-ingredient products. Among 7856 reports listing a single-ingredient product as the sole suspect, 15.8% were SAEs, including eight fatal cases. However, the causality remained unclear due to insufficient data. *Ganoderma lucidum*, *Viscum album*, and *Silybum marianum* were most frequently associated with SAEs. In multi-ingredient products, propolis was frequently linked to hypersensitivity and skin reactions. **Conclusions**: This study provides a comprehensive overview of the safety profiles of adaptogenic and immunomodulatory natural products. Variability in product composition, lack of standardization, incomplete reporting in clinical studies, and underreporting in pharmacovigilance databases complicate accurate risk assessment. For multi-ingredient products, attributing specific AEs to specific components remains difficult. Further high-quality clinical research and improved pharmacovigilance are needed, along with clear safety warnings to reduce risks for consumers.

## 1. Introduction

Natural products with purported adaptogenic and immunomodulatory properties have been used for centuries in traditional medicine systems across Eurasia. Examples are Ashwagandha and Brahmi in Ayurveda; Astragalus, Reishi and Cordyceps in Traditional Chinese Medicine (TCM); and Eleuthero in Russian folk medicine. Adaptogens are natural substances believed to enhance the body’s ability to cope with stress, improve physical and mental performance, and restore physiological balance [1]. The concept of adaptogens was first introduced in the mid-20th century by Russian researchers seeking substances that could boost resilience to various stressors [1]. In contrast, immunomodulatory natural products influence the immune system by either stimulating or suppressing specific immune responses [2].

To be classified as an adaptogen, a natural product typically must meet four key criteria [3,4]. First, it must be non-specific, meaning it should help the body resist a wide range of physical, chemical, or biological stressors. Second, it should help maintain homeostasis, keeping the body stable despite disruptions. Third, it must be safe and non-toxic, causing minimal side effects with long-term use. Fourth, it must have increased effectiveness under stress or pathological conditions, thus becoming more beneficial when the body is challenged. While these criteria provide a conceptual framework, it is important to note that the definition of an adaptogen remains somewhat ambiguous and that scientific evidence supporting many of these claims is still limited [5].

Although some studies suggest that adaptogens primarily act on the hypothalamic–pituitary–adrenal (HPA) axis, which regulates the body’s response to stress, the exact mechanisms of action are not yet fully understood [3]. It is also proposed that adaptogens enhance the body’s cellular and systemic defense mechanisms by activating both intracellular and extracellular signaling pathways [4]. These pathways upregulate the expression of stress-responsive proteins and neuropeptides, which are thought to enhance the body’s ability to manage stress and maintain homeostasis [5].

Several natural products are recognized for their adaptogenic properties in herbal monographs published by the European Medicines Agency (EMA). These include *Rhodiola rosea* L. (golden root) [6], *Withania somnifera* (L.) Dunal (Ashwagandha) [7], *Panax ginseng* C.A. Meyer (Asian ginseng) [8], and *Eleutherococcus senticosus* (Rupr. & Maxim.) Maxim. (Eleuthero or Siberian ginseng) [9]. For instance, *Rhodiola rosea* has an approved Community Herbal Monograph from the EMA, which acknowledges its potential to alleviate temporary fatigue, stress-related exhaustion, and general weakness [6]. The active compounds, rosavin and salidroside, are believed to contribute to these effects. *Panax ginseng* is also recognized in an EMA monograph for its use in reducing fatigue and improving general weakness [8]. In addition, *Echinacea purpurea* (L.) Moench (echinacea) has been extensively studied for its immunostimulatory effects, particularly during colds, and in the treatment of minor skin conditions and wounds [10]. Immunomodulatory natural products can be sub-classified into three categories based on their effects on the immune system: immunostimulants, immunosuppressants, and immunoadjuvants [2].

Immunostimulants are claimed to enhance the immune system’s activity, strengthening the body’s defenses against infections by boosting both the innate and adaptive immune responses. These agents promote the production and activation of immune cells such as macrophages, T-cells, and natural killer (NK) cells. Natural products believed to possess these properties include the roots and aerial parts of *Echinacea purpurea* and the roots of *Astragalus membranaceus*. Products of both plant species have been demonstrated to enhance immune cell activity and cytokine production, thereby improving the body’s response to infections [11]. In *Echinacea purpurea*, characteristic constituents such as alkylamides are thought to contribute to its immunostimulatory effects, with the highest concentrations found in the roots [11]. Some studies suggest that alkylamides may modulate immune function by interacting with cannabinoid receptor type 2 (CB2) [12]. Similarly, in *Astragalus membranaceus*, polysaccharides, flavonoids, and saponins, particularly astragalosides, are considered to play a role in its immunomodulatory effects. Research indicates that the polysaccharides may enhance macrophage activity and stimulate cytokine production, including interferon, interleukin-2 (IL-2), and TNF-α [13]. Additionally, astragaloside IV has been suggested to promote T-cell activation and support overall immune function [14].

Immunosuppressants are used to regulate an overactive immune system, which is crucial in managing conditions where excessive immune activity leads to chronic inflammation or autoimmune disorders, such as rheumatoid arthritis or inflammatory bowel disease. Compounds like withanolides from *Withania somnifera* and curcumin from *Curcuma longa* L. (Turmeric) have demonstrated potential in inhibiting inflammatory pathways, thereby reducing excessive immune response [15,16].

Immunoadjuvants are agents that enhance the immune response to antigens. They are particularly useful when paired with other treatments to boost immunity. While they do not directly activate the immune system, they improve the recognition and activation of immune responses when antigens are present. For example, beta-glucans found in medicinal mushrooms such as *Ganoderma lucidum* (Curtis) P. Karst (Reishi) and *Lentinula edodes* (Berk.) Pegl. (shiitake) have been studied for their potential to modulate immune responses by enhancing immune recognition. Some studies suggest that beta-glucans may improve the efficacy of immune responses, including responses to vaccines, by stimulating the activity of macrophages and other immune cells [17,18].

Several standardized extracts of adaptogenic and immunomodulatory natural products are commercially available. For instance, *Withania somnifera* extract KSM-66 is standardized to 5% withanolides [19], *Rhodiola rosea* extract SHR-5 is standardized to 3% rosavins and 1% salidroside [20], and *Panax ginseng* G115 is standardized to a total ginsenoside content of 4% [21].

While many natural products have a long history of traditional use, their safety profiles are not always well understood. A comprehensive assessment of potential AEs and interactions is essential to ensure their responsible use. This scoping review aims to systematically map the existing evidence on the safety of natural products with claimed adaptogenic and/or immunomodulatory properties. Specific attention is given to SAEs, which are defined as events that are life-threatening, require hospitalization, result in death, cause persistent or significant disability, or lead to birth defects [22]. AEs are further categorized by severity as mild (not interfering with routine activities), moderate (interfering with routine activities), or severe (making it impossible to perform routine activities). SAEs are typically classified as severe due to their substantial impact on health and daily functioning [23]. Furthermore, this study examines individual case safety reports (ICSRs) from the worldwide pharmacovigilance database VigiBase. Based on the findings, the review highlights key safety concerns and identifies areas that require further research in order to establish reliable safety guidelines for these popular natural remedies. The study is structured into sections focusing separately on single-ingredient products and multi-ingredient products.

## 2. Results

### 2.1. Scoping Review

**Figure 1:**
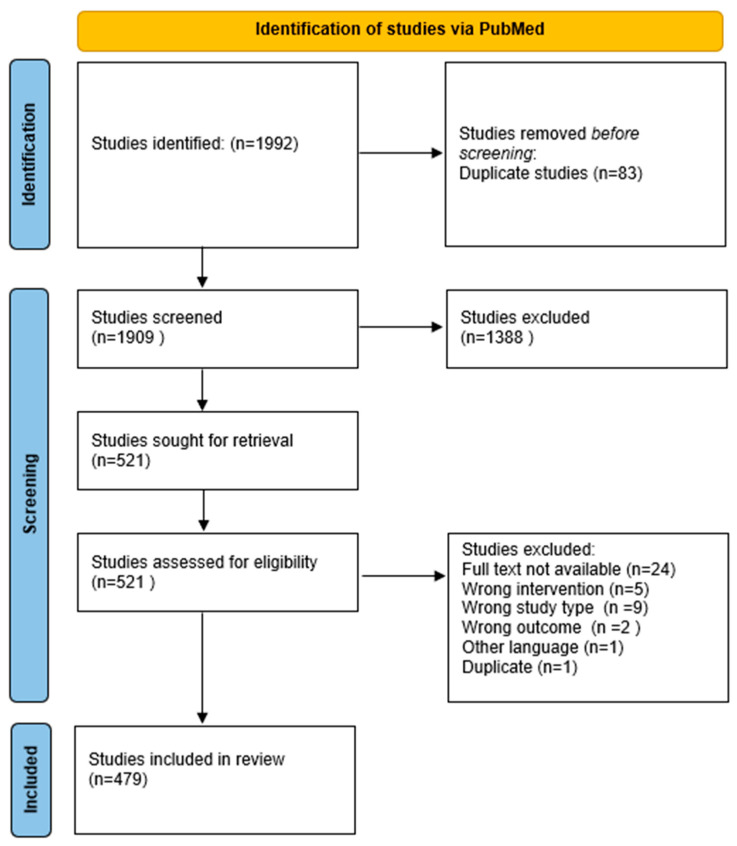
PRISMA flowchart illustrating the study selection process for the scoping review.

**Figure 2:**
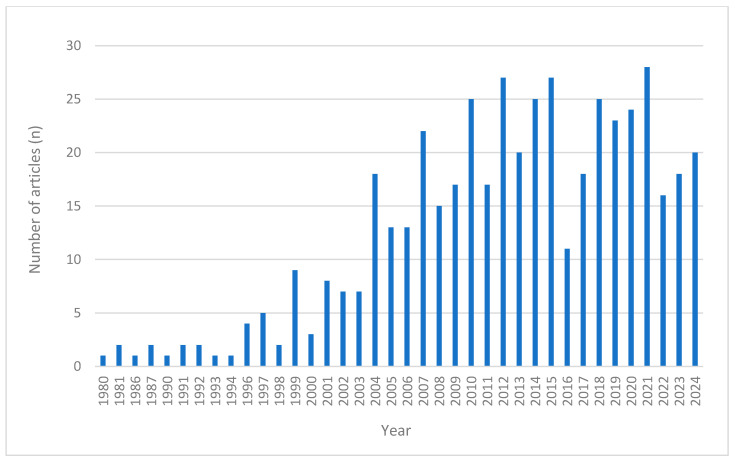
Annual number of PubMed-indexed articles (1980–2024) on the safety of natural products with adaptogenic and/or immunomodulating properties.

The scoping review identified 479 eligible studies from PubMed (Figure 1), comprising 404 studies on single-ingredient interventions and 75 studies on multi-ingredient interventions (Table 1). Of these, 339 were clinical trials and 140 were case studies. Figure 2 illustrates the annual number of publications retrieved from PubMed between 1980 and 2024, revealing a steady increase in research output, particularly from the early 2000s onward.

The ten most frequently studied natural products are listed in Table 2, with *Curcuma longa* identified as the most extensively researched, followed by *Panax ginseng* and *Zingiber officinale* Roscoe (ginger). Together, these ten products accounted for nearly 60% of all investigated natural substances. A detailed breakdown of the natural products across clinical trials is provided in Table S1.

#### 2.1.1. Single-Ingredient Products

From the scoping review, 51 natural products with adaptogenic and/or immunomodulating activities were retrieved. They were studied in a total of 285 clinical trials (Table S1). Of these, 236 were randomized, while 49 were non-randomized, all of them specifically investigating the effects of single-ingredient interventions. A total of 119 case studies were identified concerning single-ingredient products (Table 3). The most frequently reported were *Glycyrrhiza glabra* L. (licorice), *Camellia sinensis* L. Kuntze (green tea), *Lentinula edodes*, *Panax ginseng,* and *Allium sativum* L. (garlic). A detailed distribution of natural products across the case studies is provided in Table S2.

##### Adverse Events Reported in Clinical Trials

A scoping review of 285 clinical trials investigating single-ingredient natural products with adaptogenic and/or immunomodulating properties revealed a broad spectrum of reported adverse events (AEs). The most commonly observed AEs were gastrointestinal disturbances, such as diarrhea, nausea, and abdominal discomfort. These symptoms were generally mild to moderate in severity and did not lead to study discontinuation in most cases. *Curcuma longa* was the most frequently studied natural product [14,24,25,26,27,28,29,30,31,32,33,34,35,36,37,38,39,40,41,42,43,44,45,46,47,48,49,50,51,52,53,54,55,56,57,58]. Overall, the clinical trials demonstrated favorable safety profiles, with most AEs being transient and non-serious, resolving upon discontinuation of the investigational product.

Serious adverse events (SAEs) were reported in three clinical trials [59,60,61]. In a study by Taylor et al. [59], children aged 2 to 11 years were treated with *Echinacea purpurea* for up to 10 days at the onset of upper respiratory tract infection (URI) symptoms. The product used was a liquid oral syrup derived from the dried, pressed juice of the aerial parts of the plant harvested during flowering. It was not standardized to specific compounds, and no quantification of active constituents was provided. Dosage was age-dependent, with children aged 2 to 5 years receiving 7.5 mL/day (3.75 mL twice daily) and those aged 6 to 11 years receiving 10 mL/day (5 mL twice daily). A total of 337 URIs were treated with Echinacea, and 370 URIs with placebo. Two SAEs occurred in the *Echinacea purpurea* group, involving the sudden onset of stridor following administration of the study medication, requiring an emergency department visit. Both patients were treated with oral steroids as outpatients and excluded from further participation in the study. In a study by Fried et al. [60], 154 patients with chronic hepatitis C virus (HCV) infection who had failed interferon-based therapy were randomized to receive either Legalon 140 (a standardized extract of *Silybum marianum* L. Gaertn. (milk thistle) at 420 mg or 700 mg or a placebo three times daily for 24 weeks. A total of 12 SAEs were reported: one in the placebo group, six in the 420 mg silymarin group and five in the 700 mg silymarin group. The specific nature of these SAEs was not detailed. The most frequently reported non-serious AEs were gastrointestinal symptoms, affecting 12% of the silymarin group compared to 5% of the placebo group. One participant in the 420 mg silymarin group died by suicide 12 weeks after completing treatment. However, causality was deemed unlikely, as the event occurred post-treatment and other contributing factors may have been involved. Overall, the incidence of AEs was comparable between the treatment and placebo groups. In the Minnesota Green Tea Trial (MGTT) by Dostal et al. [61], 1075 postmenopausal women at high risk for breast cancer received either 1315 mg of green tea extract (GTE) containing 843 mg of epigallocatechin (EGCG) or a placebo daily for 12 months. A total of 26 SAEs were reported in 20 participants: 4 SAEs in 12 GTE recipients and 9 SAEs in 8 placebo recipients. The most frequent SAE in the GTE group was elevated alanine aminotransferase (ALT) levels, occurring in seven participants. Six cases were Grade 3 in severity, and one was Grade 4 in severity. The incidence of ALT elevations was significantly higher in the GTE group (6.7%) than in the placebo group (0.7%, *p* < 0.001). Other SAEs in the GTE group included surgical procedures (n = 3), a neoplasm (n = 1), and a gastrointestinal disorder (n = 1). Additionally, six SAEs fell into three specific categories: Two cases were related to surgical and medical procedures, two cases were related to neoplasms, and two cases were associated with injury- or poisoning-related complications. Furthermore, one SAE occurred in each of the following categories: vascular disorders (n = 1), gastrointestinal disorders (n = 1), and metabolism/nutrition disorders (n = 1). All surgical procedures in both the GTE and placebo groups were elective or linked to a pre-existing condition.

##### Adverse Events Reported in Case Studies

Several case studies (Table S2) have highlighted significant cardiovascular AEs, particularly associated with *Glycyrrhiza glabra* [62,63,64,65,66,67,68]. Prolonged licorice consumption was linked to severe hypokalemia, which in some cases triggered life-threatening cardiac complications such as Torsades de Pointes, ventricular fibrillation, and hypertensive crises. Licorice-induced pseudoaldosteronism was reported by Yoshino et al. [65], characterized by hypertension, metabolic alkalosis, and electrolyte imbalances, which led to complications such as rhabdomyolysis and generalized muscle weakness. In one case, a patient developed severe quadriparesis with creatine kinase levels exceeding 63,000 IU/L [69]. Another report described a fatal outcome involving pulmonary congestion, black stomach contents, and kidney tubular damage following prolonged licorice intake [67]. Hepatotoxicity has also been reported, particularly in association with *Curcuma longa*, *Camellia sinensis*, and *Aloe vera* [70,71,72,73,74,75,76,77,78,79,80,81,82,83,84,85,86,87,88,89,90,91,92,93]. Five cases of liver injury attributed to *Curcuma longa* ranged from transient transaminase elevations to acute hepatitis and drug-induced autoimmune hepatitis [70,71,72,73,74]. Similarly, 11 cases of severe hepatotoxicity following the use of *Camellia sinensis* have been reported, with some progressing to fulminant liver failure requiring liver transplantation [75,76,77,78,79,80,81,82,83,84,85]. *Aloe vera* has been associated with hepatocellular injury, toxic hepatitis, and intrahepatic cholestasis [86,87,88,89,90,91,92,93]. Severe allergic reactions have been reported following exposure to *Viscum album* L. (mistletoe) and *Echinacea purpurea* [94,95,96,97,98,99,100]. For *Viscum album*, AEs were primarily associated with subcutaneously injected whole-plant aqueous extracts and included localized inflammatory reactions such as panniculitis at the injection site, systemic hypersensitivity responses, and Grade II anaphylactic reactions. Immunological evaluations confirmed IgE-mediated responses in some cases, via basophil activation tests or positive intradermal skin tests. Other reported systemic symptoms included urticaria, hypotension, asthma, and facial edema. Additionally, oral preparations containing *Viscum album* in combination with other herbs have been linked to hepatotoxicity, including biopsy-confirmed hepatitis and liver necrosis [94,95,96,97,98].

Cases of *Echinacea purpurea* involved orally administered preparations derived from aerial parts, root, or whole plants. Reported AEs included anaphylaxis, new-onset asthma, and recurrent episodes of erythema nodosum [99,100]. Dermatological reactions were frequently associated with *Lentinula edodes* (shiitake mushroom), particularly following the ingestion of raw or undercooked forms. The most characteristic presentation was flagellate dermatitis, characterized by linear, whip-like erythematous streaks on the trunk, limbs, and other areas. In addition to ingestion, occupational exposure among mushroom cultivators has been associated with contact dermatitis and allergic alveolitis [101,102,103]. These diagnoses were often supported by positive skin prick tests, the detection of shiitake-specific IgE, and symptom resolution upon exposure cessation. Additionally, inhalation of spores has been implicated in hypersensitivity pneumonitis, supported by positive serum precipitins and radiological findings [101,102,103,104,105,106,107,108,109,110,111]. *Allium sativum* L. (garlic), especially in raw or crushed form, has been associated with chemical burns and irritant dermatitis. These effects occurred predominantly after prolonged topical application to treat skin conditions such as warts, acne, or fungal infections [112,113,114,115,116,117,118,119].

Neurotoxic effects have been documented for *Azadirachta indica* A. Juss. (neem oil), causing encephalopathy, optic neuropathy, and seizures, particularly in infants and young children [120,121]. In these cases, small quantities of neem oil were given orally by caregivers for perceived health benefits, such as relief from indigestion, cough, and parasitic infections. Cases of neem oil poisoning resulting from attempted suicide by overdose resulted in metabolic acidosis, hyperglycemia, renal failure, and hepatotoxicity, with MRI findings demonstrating cytotoxic edema in the basal ganglia [122,123,124].

Renal toxicity has been associated with the consumption of *Inonotus obliquus* (Ach. ex Pers.) Pilat. (Chaga mushroom) and *Momordica charantia* L. (bitter melon) [125,126,127]. Long-term consumption of Chaga mushroom powder led to end-stage renal disease due to oxalate nephropathy, confirmed by kidney biopsy findings of extensive calcium oxalate crystal deposition [125,126]. Oral supplementation of Bitter melon extract was associated with acute interstitial nephritis, with renal biopsy findings revealing tubular damage and immune cell infiltration [127].

Additional cases highlighted unexpected AEs, such as mania following ingestion of a *Panax ginseng* root extract solution (300 mg/day for two weeks) [128]. Severe hypereosinophilia accompanied by gastrointestinal symptoms has been reported following the use of *Echinacea purpurea* supplements; the exact composition, dosage, and duration of use were not specified [129].

##### Herb–Drug Interactions

Several reports have described complex herb–drug interactions with clinically significant consequences. *Viscum album* and *Curcuma longa* have been shown to inhibit cytochrome P450 enzymes CYP2C9 and CYP3A4, resulting in elevated paclitaxel concentrations and subsequent hepatotoxicity [70].

*Bacopa monnieri* (L.) Wettst. (Brahmi) was associated with cholinergic toxicity when co-administrated with cevimeline, a muscarinic receptor agonist. Reported symptoms included hyperhidrosis, nausea, dizziness, and tachycardia [130].

*Uncaria tomentosa* (Willd. ex Schult.) DC. (cat’s claw) was linked to serotonin syndrome in a patient concurrently taking serotonergic medications, and exacerbation of Parkinson’s disease symptoms was reported in another case [131,132,133].

The use of *Eleutherococcus senticosus* (Rupr. & Maxim.) Maxim. (Eleuthero or Siberian ginseng) resulted in elevated serum digoxin levels, possibly due to interference with drug metabolism or assay cross-reactivity [134].

*Cordyceps sinensis* (Berk.) Sacc. has been associated with prolonged bleeding following dental surgery, likely due to its anticoagulant properties [135].

Herb–drug interactions may occur through pharmacokinetic or pharmacodynamic mechanisms [136].

#### 2.1.2. Multi-Ingredient Products

##### Adverse Events Reported in Clinical Trials

A total of 54 clinical trials assessed the safety of multi-ingredient formulations with adaptogenic and/or immunomodulating properties. Of these, 47 were randomized controlled, while 7 were non-randomized. Frequently studied combinations included *Zingiber officinale* (six studies), *Echinacea purpurea* (five studies), and *Silybum marianum* (five studies). Table S1 provides a detailed overview of the number of studies involving multi-ingredient products. No SAEs were reported in any of these clinical trials. Among the 54 studies, 20 reported no AEs, while the remaining 34 studies documented only mild to moderate AEs.

##### Adverse Events in the Case Studies

Across the 21 case studies on multi-ingredient products, AEs were primarily associated with hepatotoxicity (7 studies, 38.1%), hypersensitivity reactions and anaphylaxis (5 studies, 23.8%), dermatological reactions (5 studies, 23.8%), renal toxicity (2 studies, 9.5%), neurological and psychiatric effects (1 study, 4.8%), and cardiovascular events (1 case, 4.8%). Among these, 10 case studies (47.6%) involved formulations containing propolis [136,137,138,139,140,141,142,143,144,145].

Hepatotoxicity was the most frequently reported AR among multi-ingredient products [146,147,148,149,150,151,152]. For example, Tremlett et al. [147] reported symptomatic liver injury in a 38-year-old woman with relapsing-remitting multiple sclerosis after taking capsules containing *Azadirachta indica* and *Momordica charantia*, with an ALT level of 886 U/L and an AST level of 406 U/L. Gilbert et al. [148] described acute liver failure requiring transplantation in a 43-year-old woman who consumed a polyherbal formulation containing 12 herbs, including *Astragalus.* Teschke and Bahre [149] described severe hepatotoxicity in a 64-year-old woman using multiple Ayurvedic products, including *Bacopa monnieri*, leading to jaundice and enzyme elevations, with ALT reaching 601 U/L and aspartate aminotransferase (AST) at 663 U/L (normal reference ranges are typically ≤40 U/L for ALT and ≤35 U/L for AST, depending on the laboratory). Koenig et al. [150] described cholestatic liver injury in a 53-year-old woman after taking herbal supplements containing *Curcuma longa*, *Silybum marianum*, *Schisandra chinensis*, and *Berberis vulgaris*, with a RUCAM (Roussel Uclaf Causality Assessment Method) score of +6 indicating probable causality. Karousatos et al. [151] presented three cases of acute hepatocellular injury in women consuming Ayurvedic formulations containing *Tinospora crispa*, *Zingiber officinale*, and *Terminalia chebula* with ALT levels ranging from 760 U/L to 1382 U/L. Jiménez-Encarnación et al. [153] reported a case of severe hepatocellular necrosis in a 45-year-old woman consuming Euforia, a multi-herbal beverage containing *Camellia sinensis*, *Aloe vera*, *Lycium barbarum*, *Morinda citrifolia,* and *Curcuma longa*. Kim et al. [152] reported acute liver injury in a 55-year-old male after consuming *Viscum album* and *Pueraria lobata* (Kudzu) extracts, which were taken frequently for 1 month and 10 days, respectively. The patient experienced significantly elevated liver enzymes (AST: 1108 IU/L, ALT: 1528 IU/L) and symptoms of acute hepatitis, including fever, brown urine, and pain. Causality assessment using the RUCAM method indicated “probable” causality. Renal toxicity was observed in two studies [143,153]. Li et al. [144] described acute renal failure in a 59-year-old male following the ingestion of a propolis solution, with worsening renal function upon rechallenge. Abdul et al. [154] described a case of lupus nephritis in a 29-year-old woman who developed severe proteinuria of 10 g/24 h and hypoalbuminemia after taking “Super Kidney”, a supplement containing *Panax ginseng*, and other supplements for six months.

Hypersensitivity reactions and anaphylaxis have been reported in five cases [137,138,140,142,145]. Callejo et al. [138] documented a severe systemic allergic reaction in a 10-year-old boy with a known sensitivity to bee products who developed angioedema, urticaria, and hypotension after propolis exposure. Freedman et al. [139] reported systemic allergic dermatitis in a 32-year-old woman using oral and topical propolis, which resolved after corticosteroid treatment. McNamara and Pien [146] described a case of exercise-induced anaphylaxis in a 40-year-old male after taking bee pollen two hours before strenuous exercise. Skin testing confirmed sensitization. Bellegrandi et al. [141] documented an anaphylactic-like reaction in a 40-year-old HIV-positive woman who developed fever, dyspnea, and perioral eczema after taking a propolis solution. Ramien and Pratt [143] described a fixed drug eruption linked to propolis ingestion, presenting as a recurrent dull-red edematous plaque on the penis, which reappeared upon re-exposure.

Dermatological reactions were observed in five studies, mainly linked to propolis-containing products, either used topically or taken orally [136,139,141,144,154]. Teraki and Shiohara [137] described granulomatous contact dermatitis in a 53-year-old male using a 20% propolis lotion, with patch testing confirming propolis hypersensitivity. Silvani et al. [140] reported two cases of allergic contact dermatitis in men with psoriasis using propolis-containing creams, with positive patch test results for propolis and colophony. Brailo et al. [142] described oral erosions in a 20-year-old woman with recurrent aphthous ulcers following self-administered topical propolis use. Palanisamy et al. [155] described a photosensitivity reaction in a 32-year-old woman taking Metabolife 356, an herbal weight-loss supplement containing ginseng, goldenseal, and bee pollen. Symptoms were localized to sun-exposed skin and resolved after discontinuation and corticosteroid therapy. Angelini et al. [145] reported a case of allergic contact dermatitis in a 31-year-old female teacher with a 10-year history of relapsing psoriasis. The patient had been using two topical creams containing propolis for a month, which led to the development of red vesicular dermatitis on and around the application sites. Patch testing revealed a strong positive reaction to propolis, while the dermatitis resolved upon discontinuing the creams.

Neurological and psychiatric effects were observed in one case [156]. Yadav et al. [156] documented acute psychosis in a 42-year-old woman taking multivitamins and herbal supplements containing ginseng, goji, spirulina, and bee pollen. The patient presented with severe behavioral changes, hallucinations, and memory loss, with the Naranjo Adverse Drug Reaction Probability Scale indicating a possible causal link to supplement use.

One cardiovascular event has been reported [157]. Yigit and Cevik [157] described pulmonary embolism in a 41-year-old woman following a high-dose ingestion of 15 Panax pills containing *Tribulus terrestris*, *Avena sativa*, and *Panax ginseng*. She developed sudden dyspnea, sweating, and loss of consciousness, requiring selective pulmonary artery thrombolysis. The exact composition of the product was not disclosed in the article.

### 2.2. Analysis of Reported Adverse Events from the Global ICSR Database VigiBase

**Figure 3:**
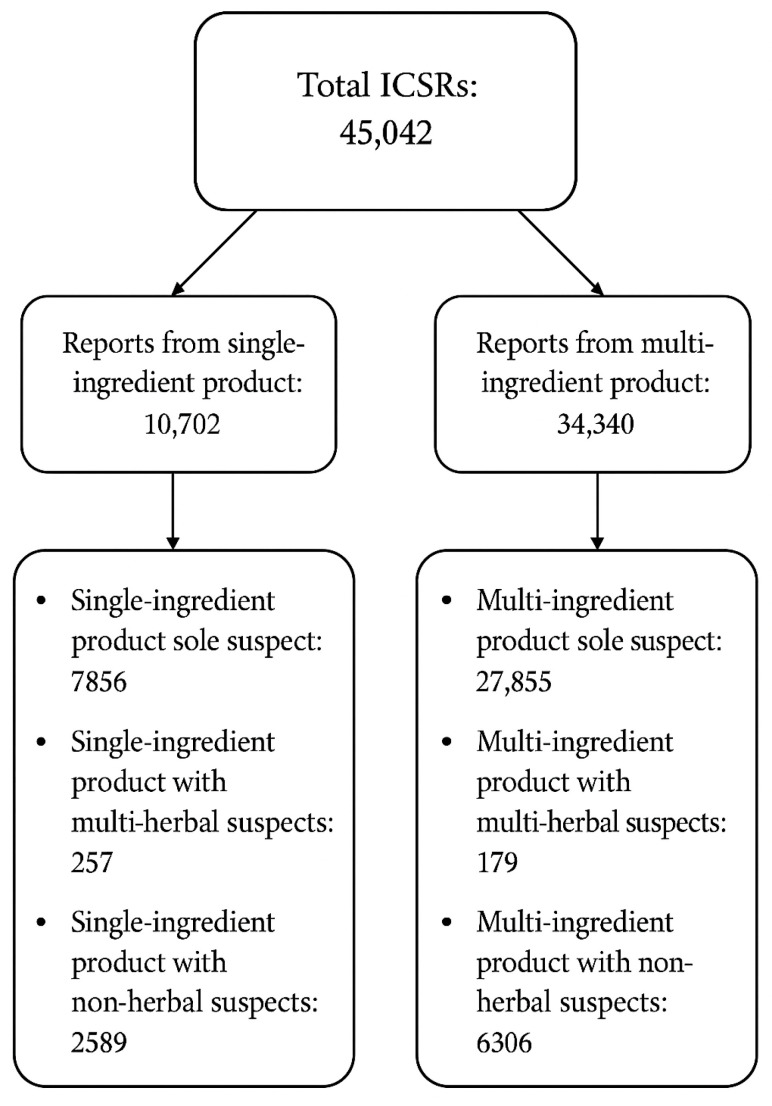
Overview of the distribution of the ICSRs in the WHO-UMC database VigiBase.

The WHO-UMC global pharmacovigilance database, VigiBase, recorded a total of 45,042 individual case safety reports (ICSRs) associated with natural products with adaptogenic and/or immunomodulating properties. Of these, 10,702 ICSRs involved single-ingredient products, while 34,340 involved multi-ingredient products. Within both categories, three subgroups were identified: (1) reports in which the natural product was the sole suspected cause of the AE, (2) reports involving multiple natural products as suspects, and (3) reports in which the natural product was combined with non-herbal products (Figure 3).

Table 4 presents the age and sex distribution of the ICSRs for both single-ingredient and multi-ingredient natural products. The majority of the reports (58.2%) originated from female users, with males contributing 39.1%. The most frequently represented age group was 45–64 years, comprising 35.3% of all ICSRs.

Table 5 presents the distribution of ICSRs for single-ingredient and multi-ingredient products across various WHO regions. The Western Pacific Region reported the highest number of ICSRs for single-ingredient products (44.7%), followed by the European Region (33.6%). Similarly, for multi-ingredient products, the Western Pacific Region accounted for the largest share of ICSRs (85.4%), while the European Region contributed 9.4%.

#### 2.2.1. Single-Ingredient Products

A total of 10,702 ICSRs involved the use of a single-ingredient product. Among these, 7856 reports identified the single-ingredient product as the sole suspected cause of the AE. Additionally, 257 reports involved multiple natural products as suspects, and 2589 reports cited a combination of the natural product and non-herbal products as potential suspects.

Table 6 presents the ten most frequently reported single-ingredient products identified as the sole suspect in ICSRs. *Ganoderma lucidum* and *Viscum album* were the most commonly reported species, followed by *Silybum marianum*. A detailed overview of these reported single-ingredient products is provided in Table S3.

AEs were classified according to System Organ Class (SOC) and Preferred Term (PT). Table S4 summarizes the reported SOCs and their corresponding PTs for the top 15 most frequently reported natural products. For *Ganoderma lucidum*, the most frequently affected SOC was general disorders and administration site conditions, with chills, chest pain and pyrexia being the most frequently reported PTs. This was followed by skin and subcutaneous tissue disorders, with pruritus, rash, erythema, and hyperhidrosis as the most frequently reported PTs. The next most common SOC was gastrointestinal disorders, with nausea, vomiting, and abdominal pain as the predominant PTs.

In the case of *Viscum album*, general disorders and administration site conditions were the most frequently reported SOC, with injection site inflammation being the predominant PT. Skin and subcutaneous tissue disorders were also common, primarily presenting as pruritus, rash, and urticaria. Gastrointestinal disorders were less frequently reported but included events such as nausea and diarrhea.

For *Silybum marianum*, gastrointestinal disorders represented the most frequently reported SOC, with diarrhea and abdominal discomfort as the most common PTs. Skin-related AEs, including pruritus and rash, were also notable. Nervous system disorders were less frequently reported, predominantly featuring dizziness and headache.

Among the ICSRs where single-ingredient products were identified as the sole suspected causative agent (n = 7856), 15.8% (n = 1242) were classified as serious (Table 7). There were eight fatal cases reported: six involved *Pelargonium sidoides*, one *Viscum album*, and one *Silybum marianum*. Two fatal cases of pancreatic carcinoma were reported in the European Region following the use of *Pelargonium sidoides*. In one case, no causality was provided; in the other, the reporter considered the event “possibly related”, while the manufacturer deemed it “unlikely related”. A case from the South-East Asia Region described sudden infant death syndrome in an infant exposed to *Pelargonium sidoides*, with no causality assessment recorded. Another South-East Asia Region report involved a woman who experienced abdominal pain and blood in stool after receiving *Viscum album*; both events were assessed as “unlikely related”.

In the European Region, a fatal case involved a toddler who received *Pelargonium sidoides* syrup for pseudocroup; vomiting and off-label use were listed as AEs and classified as “unlikely related”. A fatal pediatric case from the African Region, involving bottle feeding with *Pelargonium sidoides*, also lacked causality information. A consumer in the European Region reported the death of her husband while using *Silybum marianum*; the reporter classified this as “possible”, while the manufacturer considered it “unassessable”. Lastly, a case from the Region of the Americas described multiple AEs linked to *Pelargonium sidoides*, including death, unexpected therapeutic response, and off-label use. The cause of death was deemed “unclassifiable”, while other events were recorded as “reaction abated.”

It is important to note that these classifications were made by the respective reporters, pharmacovigilance centers, or marketing authorization holders (MAHs) submitting the reports, rather than by the UMC, and that such information is not consistently available for all cases. Table S5 provides an overview of reported SOCs and their respective PTs for the top 15 most frequently reported natural products in serious case reports.

The most reported natural product in the serious ICSRs was *Ganoderma lucidum*. The SOC general disorders and administration site conditions were the most affected, with PTs including chills, hyperpyrexia, pyrexia, and chest pain being the most common. Other notable SOCs included immune system disorders, with anaphylactoid reactions, anaphylactic shock, and hypersensitivity reported. Respiratory, thoracic, and mediastinal disorders were also commonly observed, with dyspnea and tachypnea as prominent PTs.

The second most frequently reported natural product in serious case reports was *Pelargonium sidoides*. The SOC skin and subcutaneous tissue disorders were most affected, featuring PTs such as rash, pruritus, erythema, and urticaria. Hepatobiliary disorders were also notable, with jaundice, drug-induced liver injury, hepatitis, and hepatotoxicity indicating potential hepatotoxicity. Respiratory, thoracic, and mediastinal disorders were frequently reported as well, particularly dyspnea and epistaxis.

The third most commonly reported natural product in serious cases was *Salvia miltiorrhiza*. Skin and subcutaneous tissue disorders were the most frequently occurring SOC, with AEs such as pruritus, rash, drug eruption, and erythema. General disorders and administration site conditions were also common, particularly chest pain, chills, pyrexia, and hyperpyrexia. Respiratory, thoracic, and mediastinal disorders were observed, including symptoms such as dyspnea, tachypnea, and a sensation of suffocation.

#### 2.2.2. Multi-Ingredient Products

A total of 34,340 ICSRs involved the use of multi-ingredient products. Of these, 27,855 reports identified the multi-ingredient product as the sole suspect responsible for the AE. Additionally, 179 reports involved multiple herbal products as suspects, while 6306 reports included a combination of the multi-ingredient product and non-herbal products as suspected contributors (Figure 3).

Table 8 presents the ten most frequently reported natural products from multi-ingredient products identified as the sole suspect in ICSRs. *Salvia miltiorrhiza* was by far the most frequently reported, followed by *Glycyrrhiza glabra* and *Echinacea purpurea*. The detailed overview of all reported multi-ingredient products is provided in Table S6.

Table S7 summarizes the reported SOCs and their respective PTs for the top 15 most frequently reported natural products as sole suspects in multi-ingredient products. For *Salvia miltiorrhiza*, the most frequently reported SOC was gastrointestinal disorders, with nausea, vomiting, and abdominal pain being the most common PTs. This was followed by general disorders and administration site conditions where chest pain, chills, and pyrexia were predominant PTs. Nervous system disorders were also commonly reported, particularly dizziness, headache, and hypoaesthesia.

For *Echinacea purpurea*, the most frequently reported SOC was skin and subcutaneous tissue disorders with pruritus, rash and urticaria as the primary PTs. Nervous system disorders, including ageusia, dysgeusia, and dizziness, were also common, alongside gastrointestinal disorders, including nausea and vomiting. For *Glycyrrhiza glabra*, gastrointestinal disorders were the most frequently reported SOC, with nausea, vomiting, diarrhea, and upper abdominal pain as the most common PTs. This was followed by general disorders and administration site conditions, including drug ineffectiveness, malaise, and fatigue. Skin and subcutaneous tissue disorders, particularly rash and pruritus, were also frequently reported.

## 3. Discussion

The use of natural products claiming adaptogenic and/or immunomodulatory properties has gained increasing attention due to their potential benefits in supporting immune function, stress adaptation, and overall well-being. However, concerns regarding their safety remain, particularly in relation to the occurrence of AEs. The present study provides a comprehensive evaluation of the safety profile of these products through a scoping review of the literature covering the period from 1980 to 2024. Additionally, ICSRs associated with the use of single-ingredient and multi-ingredient products were systematically analyzed. These reports were sourced from VigiBase, the global pharmacovigilance database of the WHO-UMC, which offers an extensive collection of ICSRs related to herbal product use worldwide. It is important to note that the term “natural products” encompasses a broad range of sources, including not only plants but also fungi (e.g., mushrooms) and animal-derived substances (e.g., propolis). To maintain consistency throughout this review, we have used the term “adverse event” instead of alternating with terms such as “adverse reaction”. However, it is important to note that many of the original studies may have employed the term adverse reaction.

A fundamental challenge in the scientific assessment of these natural products is the lack of clear, universally accepted definitions for “adaptogenic” and “immunomodulating”. These terms are widely used in marketing and traditional medicine claims but lack precise biochemical and pharmacological characterization [158]. Adaptogens, for example, are broadly described as substances that enhance the body’s resistance to stressors and help restore homeostasis; however, their mechanisms of action and methods of measurement remain poorly defined. Regulatory authorities such as the U.S. Food and Drug Administration (FDA) do not recognize the term “adaptogen” in pharmacological or clinical contexts. Similarly, the European Medicines Agency (EMA) refrains from using the term “adaptogen” in its herbal monographs, with the exception of *Eleutherococcus senticosus* (Rupr. et Maxim.) Maxim., radix, which is described as being traditionally used as an adaptogen [159].

The FDA has issued multiple warning letters to companies marketing dietary supplements with unapproved health claims related to adaptogenic and immunomodulatory effects. For example, in 2023, Lone Star Botanicals Inc. received a warning letter for promoting products with unsubstantiated therapeutic claims, classifying them as unapproved new drugs under the Federal Food, Drug, and Cosmetic Act [160].

While the term “immunomodulator” is better established in scientific literature, its application to natural products also presents challenges. Natural products claiming immunomodulatory effects are often marketed as supporting immune health or enhancing immune function, particularly during cold and flu seasons or for general wellness. Yet, the mechanistic data supporting these claims are frequently derived from in vitro or animal studies and may not translate to clinically meaningful outcomes in humans. Regulatory agencies, including the FDA, have expressed concern over unproven immunomodulatory claims. In 2020, Red Moon Herbs was warned for marketing products with unproven claims related to immune enhancement and disease prevention, including for COVID-19 [161]. The vagueness of the terms “adaptogenic” and “immunomodulating” likely contributes to the limited availability of high-quality scientific studies assessing the safety and clinical efficacy of these products.

### 3.1. Single-Ingredient Products

#### 3.1.1. Adverse Events Reported in Clinical Trials from the Scoping Review

The clinical trials included in the scoping review often lacked detailed information on the occurrence and nature of AEs. Most studies focused primarily on efficacy outcomes, such as immune function markers or stress-reduction parameters, while providing incomplete or inconsistent reporting of AEs. In many cases, essential details such as AE severity, duration, dose dependency, or the presence of potential confounding factors were omitted. Some studies described AEs merely as “mild” or “well tolerated” without clarifying whether they resolved spontaneously, required medical intervention, or led to treatment discontinuation. Another key limitation was the lack of standardization and detailed qualitative and quantitative characterization of the natural products assessed. Critical information, such as the specific plant part used, extraction method, and preparation, was often incomplete or ambiguously reported. This lack of clarity significantly impedes the ability to evaluate product safety and compare outcomes across studies. Furthermore, the lack of regulatory oversight for many natural products raises concerns about quality assurance. Unlike conventional medicines, natural products are not always subject to rigorous quality control by regulatory authorities. As a result, formulations may contain undeclared plant species, contaminants, or adulterants. Without standardization and concomitant quality assurance of the products, meaningful cross-study comparisons will become difficult if not impossible.

Additionally, most of the AE data from the scoping review were derived from case studies. Unlike clinical trials, which are designed to systematically monitor and document all potential AEs, case reports are typically published only in response to notable or severe events. This selective reporting introduces potential bias, possibly overrepresenting rare but dramatic AE while underreporting more common, mild, or transient reactions. Consequently, the available data may not accurately reflect the true incidence, nature, or severity spectrum of AEs associated with these natural products.

#### 3.1.2. Serious Adverse Events Reported in Clinical Trials Retrieved in the Scoping Review

Although the overall safety profiles of the single-ingredient natural products evaluated in the clinical trials were generally favorable, with most AEs being mild and transient, three clinical trials reporting SAEs raise concerns about potential risks, especially in vulnerable populations. These studies involved *Echinacea purpurea*, *Silybum marianum*, and *Camellia sinensis* and are discussed in detail below.

The study on *Echinacea purpurea* conducted by Taylor et al. [59] illustrates the potential for immune-modulating herbs to trigger hypersensitivity reactions. The two cases of severe stridor following administration of oral liquid syrup made from the pressed juice of the aerial part of *Echinacea purpurea* suggest that the herb may provoke airway inflammation or immune-mediated reactions, particularly in children. The findings by Mullins and Heddle [100] further support that *Echinacea purpurea* can induce IgE-mediated allergic responses, particularly in atopic individuals. As a member of the Asteraceae family, a group known for its allergenic potential, *Echinacea purpurea* is likely to induce reactions via immune mechanisms involving IgE production, mast cell degranulation, and histamine release. These processes may lead to symptoms such as airway inflammation, bronchospasm, and anaphylaxis [100,161].

The trial on *Silybum marianum* by Fried et al. [60] raises questions about its long-term safety in individuals with pre-existing liver conditions. Although milk thistle is widely recognized for its hepatoprotective properties, its use in patients with chronic hepatitis C was associated with 12 SAEs, including gastrointestinal disturbances and, notably, one case of suicide. However, a major limitation of the study is the lack of detailed characterization of the SAEs, making it unclear whether these events were directly attributable to *Silybum marianum* or were consequences of disease progression or other factors.

The Minnesota Green Tea Trial (MGTT) by Dostal et al. [61] raised concerns regarding the potential hepatotoxicity of high-dose green tea extract (GTE) supplementation. Participants in the GTE group experienced a significantly higher incidence of elevated ALT levels compared to placebo (6.7% vs. 0.7%), suggesting that excessive intake of epigallocatechin gallate (EGCG), the primary catechin in green tea, may lead to liver enzyme abnormalities. These findings align with other case reports documenting green tea-associated liver injury. EGCG undergoes extensive hepatic metabolism, and its accumulation at high doses may induce oxidative stress on hepatocytes, contributing to liver damage [162,163]. In addition to elevated ALT levels, several other SAEs were reported in the GTE group, including surgical procedures, neoplasms, and metabolic disorders. However, five participants who experienced these SAEs had pre-existing conditions, exhibited symptoms prior to randomization, or had discontinued the study product more than four months before the SAE occurred, complicating the assessment of causality. Additionally, each SAE related to elevated ALT levels also involved at least one confounding factor, such as concurrent infections, new medications, alcohol consumption, or a self-reported history of liver enzyme abnormalities. The dose used in this trial (1315 mg GTE containing 843 mg EGCG per day, equivalent to four cups of green tea) exceeds typical Western consumption but falls within the range reported in some Asian populations. The dosing regimen was based on habitual green tea intake in regions such as Japan and China, where daily consumption of multiple cups is common. However, despite similarities in total catechin intake, differences in bioavailability, metabolism, and mode of administration (capsule vs. brewed tea) may influence the observed hepatotoxic effects and limit the generalizability of these findings to traditional green tea consumption.

#### 3.1.3. Adverse Events Reported in Case Studies Retrieved in the Scoping Review

The case reports included in the scoping review revealed AEs across multiple SOCs, including cardiovascular, hepatic, renal, dermatological, and immunological complications. A particularly notable concern is the cardiotoxicity of *Glycyrrhiza glabra*. Several cases included severe hypokalemia, hypertensive crises, and life-threatening arrhythmias such as Torsades de Pointes and ventricular fibrillation [17,18]. The underlying mechanism, known as licorice-induced pseudoaldosteronism, results from the inhibition of 11β-hydroxysteroid dehydrogenase type 2 (11β-HSD2), leading to excessive mineralocorticoid receptor activation [164]. This imbalance causes potassium depletion, sodium retention, and metabolic alkalosis, ultimately predisposing individuals to cardiac events. 

Hepatotoxicity was another frequently reported AE, particularly associated with *Curcuma longa*, *Camellia sinensis*, and *Aloe vera*. While *Curcuma longa* is widely known for its anti-inflammatory effects, several cases of *Curcuma longa*-induced liver injury have been reported, ranging from transient elevations in liver enzymes to acute hepatitis and drug-induced autoimmune hepatitis [70,71,72,73,74]. The mechanisms underlying this hepatotoxicity are likely multifactorial. Some cases of drug-induced autoimmune hepatitis suggest that curcumin may act as a trigger for immune dysregulation, leading to a loss of immune tolerance and subsequent hepatocyte damage. Histopathological findings, such as plasma cell infiltration and periportal inflammation, support this hypothesis. These AEs were observed following a range of exposure durations, from several days to many months, and often involved high daily doses (e.g., one case described a dose of approximately 20–30 pills per day, without further details about the dose) or prolonged intake of *Curcuma longa* supplements [70].

*Camellia sinensis* extract has also been linked to hepatotoxicity, including fulminant liver failure requiring transplantation. The hepatotoxic effects are primarily attributed to epigallocatechin gallate (EGCG), the most abundant catechin in green tea, which at high doses can induce oxidative stress, disrupt mitochondrial function, and disrupt hepatocyte survival [75,76,77,78,79,80,81,82,83,84,85].

Similarly, *Aloe vera*-induced hepatotoxicity, including hepatocellular injury, toxic hepatitis, and intrahepatic cholestasis, is primarily attributed to its anthraquinone constituents, particularly aloin and emodin [165]. Upon metabolic conversion to reactive anthrones, these compounds may induce oxidative stress and mitochondrial dysfunction in hepatocytes. Reports of *Aloe vera*-related liver injury typically describe a hepatocellular pattern of damage, with clinical presentations resembling acute viral hepatitis [158].

Severe allergic and immunological reactions to *Viscum album* have been reported, all of which involved subcutaneous injections [94,95,96,97,98,99,100]. *Viscum album* extracts contain lectins (Viscumin or ML-I) that stimulate the immune system by binding to carbohydrate structures on immune cells, leading to the release of pro-inflammatory cytokines such as IL-1β, TNF-α, and IL-6. However, in sensitized individuals, this immune activation may shift to an IgE-mediated hypersensitivity response, leading to systemic mast cell degranulation, histamine release, and anaphylaxis. Basophil activation tests confirmed these exaggerated responses in affected patients. Importantly, these cases highlight the risks associated with parental administration of herbal products. Especially in Germany, subcutaneous injections are sometimes used by Heilpraktiker (alternative practitioners) for alternative cancer treatment. It is important to note that this practice falls outside the EMA definition of phytotherapy, which prohibits injectable administration of herbal medicines [166].

Dermatological effects were primarily associated with the consumption of raw or undercooked *Lentinula edodes* and topical exposure to *Allium sativum*. *Lentinula edodes*-induced flagellate dermatitis manifests as whip-like, linear erythematous streaks across the skin. This reaction is attributed to lentinan, a β-glucan with potent immunomodulatory effects [167,168]. Lentinan stimulates macrophages and dendritic cells via dectin-1 and Toll-like receptors (TLRs), leading to a cytokine cascade involving TNF-α and IL-6. The delayed onset of the rash, typically 24–48 h after shiitake consumption, suggests an immune-mediated mechanism rather than direct toxicity. Occupational exposure, particularly among cultivators of shiitake mushrooms, also led to contact dermatitis and allergic alveolitis, likely caused by airborne fungal spores or β-glucan-mediated immune activation in respiratory epithelial cells.

*Allium sativum*-induced irritant dermatitis and chemical burns have been reported primarily in the context of topical exposure to raw garlic, either through intentional application in home remedies or accidental skin contact during food preparation [112,113,114,115,116,117,118,119]. The topical use of garlic is driven by its proposed antimicrobial, antifungal, and anti-inflammatory effects for treating certain skin conditions. The injuries result from the high concentration of sulfur-containing compounds, particularly allicin and diallyl disulfide. These compounds exhibit antimicrobial and cytotoxic properties, but prolonged skin exposure can lead to direct keratinocyte damage by disrupting cellular membranes and inducing oxidative stress. Allicin can penetrate the epidermal barrier, forming reactive oxygen species (ROS) that trigger lipid peroxidation, mitochondrial dysfunction, and apoptosis in skin cells. Histological examination of affected skin in severe cases revealed epidermal necrosis, consistent with second-degree burns.

Neurotoxicity associated with *Azadirachta indica* (neem oil) has been reported, particularly in infants and young children. This is linked to constituents, such as azadirachtin and limonoids, which disrupt both neuronal and mitochondrial function. Most exposures are accidental, occurring via nasal or oral administration of traditional remedies for coughs, colds, abdominal pain, and deworming [120,121]. However, neem oil poisoning has also been documented in suicide attempts in adults [122,123,124]. Azadirachtin has been shown to inhibit mitochondrial complex I, leading to impaired oxidative phosphorylation and ATP depletion. This results in neuronal energy failure, increased oxidative stress, and eventual cytotoxic edema, particularly in metabolically active regions such as the basal ganglia, as evidenced by MRI findings.

Renal toxicity associated with *Inonotus obliquus* and *Momordica charantia* is primarily mediated by oxalate accumulation and immune-mediated nephrotoxicity, respectively. *Inonotus obliquus* (Chaga mushroom) contains exceptionally high levels of oxalates, which, when chronically consumed, may precipitate as calcium oxalate crystals in renal tubules, leading to nephrolithiasis and obstructive nephropathy [125,126]. Kidney biopsy findings in affected individuals revealed extensive tubular injury, inflammation, and interstitial fibrosis, indicative of progressive oxalate nephropathy. Given that oxalate metabolism is influenced by dietary intake, hydration status, and genetic factors, individuals with predisposing conditions such as chronic kidney disease or a history of kidney stones may be at heightened risk for *Inonotus obliquus*-induced renal failure [169].

*Momordica charantia* (bitter melon) extract has been linked to acute interstitial nephritis, likely driven by immune-mediated mechanisms. Renal biopsies revealed tubular damage, mononuclear cell infiltration, and interstitial edema, consistent with an inflammatory etiology [127].

#### 3.1.4. Herb–Drug Interactions

Herb–drug interactions present a significant area of concern, as highlighted in several reports in the scoping review. They occur when the pharmacological effects of an herbal product alter the action of a concurrently administered medication and can be pharmacokinetic or pharmacodynamic. Pharmacokinetic interactions affect the drug’s absorption, distribution, metabolism, or excretion. Most common is modulation (inhibition or induction) of drug-metabolizing enzymes such as the cytochrome P450 isoenzymes of drug transporters like P-glycoprotein. Pharmacodynamic interactions influence the drug’s target site or physiological response and may lead to additive, synergistic, or antagonistic outcomes [136]. Both types of interactions can result in a reduced therapeutic efficacy or an increased risk of toxicity, particularly if a patient takes medicines with a narrow therapeutic index. The likelihood and severity of herb–drug interactions depend on factors such as the dose and duration of herbal product use (exposure), patient-specific characteristics (e.g., co-morbidities, genetic variations), and the therapeutic index of the medication involved. In some cases, herb–drug interactions may contribute directly to AEs, underscoring the importance of identifying them and understanding the impact on clinical practice. Regularly, the use of herbal products by a patient taking prescription drugs is not disclosed to the healthcare provider. This unmonitored use may increase the likelihood of unforeseen AEs or therapeutic failure.

One of the most notable pharmacokinetic interactions involved the inhibition of CYP2C9 and CYP3A4 by *Viscum album* and *Curcuma longa*, which resulted in elevated systemic concentrations of paclitaxel and subsequent hepatotoxicity [70]. In this case, the patient undergoing chemotherapy with paclitaxel and carboplatin developed acute toxic hepatitis after concurrently using oral *Curcuma longa* (15 g/day) and *Viscum album* (13.5 mg/day), beginning four days after the first chemotherapy cycle. Curcuminoids, the bioactive polyphenols in *Curcuma longa*, have been shown to inhibit CYP enzymes in vitro, particularly CYP3A4, which is responsible for metabolizing a wide range of pharmaceutical drugs, including paclitaxel [170,171]. Inhibition of CYP3A4 reduces paclitaxel clearance, potentially leading to accumulation and hepatotoxic effects [172]. The patient’s hepatotoxicity was exacerbated by additional exposure to a contaminated Chlorella supplement, which contained microcystin-LR, a potent hepatotoxin produced by cyanobacteria. The accumulation of paclitaxel, combined with direct hepatotoxic effects from the microcystin, likely contributed to the severity of the liver damage.

Another noteworthy pharmacokinetic interaction involved *Eleutherococcus senticosus* and digoxin. *Eleutherococcus senticosus* contains eleutherosides, glycosides that are structurally similar to cardiac glycosides such as digoxin [173]. In the reported case, a patient with previously stable digoxin levels experienced a significant, unexplained increase in serum digoxin after initiating Eleutherococcus supplementation [134]. The patient’s digoxin dosage remained unchanged, yet elevated levels persisted even after temporary cessation of digoxin. Only upon discontinuation of Eleutherococcus digoxin levels returned to baseline. A subsequent rechallenge with *Eleutherococcus* led to another increase in digoxin levels, which again normalized upon stopping the supplement. Forensic analysis ruled out contamination with digoxin or digitoxin, suggesting that a constituent of *Eleutherococcus senticosus* may inhibit digoxin metabolism or elimination, leading to elevated systemic levels and increased toxicity risk.

In terms of pharmacodynamic interactions, *Bacopa monnieri* demonstrated cholinergic toxicity when combined with cevimeline, a muscarinic receptor agonist used to treat dry mouth in Sjögren’s syndrome. *Bacopa monnieri* contains bacosides, which exert cholinergic effects through acetylcholinesterase (AChE) inhibition and enhanced synaptic acetylcholine levels [174,175]. This mechanism is similar to that of cevimeline, leading to an additive cholinergic effect and an increased risk of AEs such as hyperhidrosis, nausea, dizziness, and tachycardia.

Another significant pharmacodynamic interaction involved *Uncaria tomentosa* (cat’s claw) and serotonergic medications such as fluoxetine or venlafaxine. *Uncaria tomentosa* contains alkaloids, which have been shown to modulate serotonin receptors and inhibit serotonin reuptake [176]. When combined with selective serotonin inhibitors (SSRIs) or serotonin–norepinephrine reuptake inhibitors (SNRIs), this may lead to serotonin syndrome, characterized by symptoms such as agitation, diaphoresis, tremor, and confusion due to excessive serotonergic activity.

Finally, the anticoagulant properties of *Cordyceps sinensis* were implicated in prolonged bleeding after dental surgery. *Cordyceps sinensis* contains nucleosides, polysaccharides, and cordycepin, a 3′-deoxyadenosine derivative, which have been shown to inhibit platelet activation and reduce clot formation by modulating adenosine pathways and suppressing thromboxane A2 synthesis [177,178]. These effects may impair clot formation and prolong bleeding time, posing a significant risk in patients undergoing surgical procedures or those on concurrent anticoagulant therapy.

#### 3.1.5. Analysis of Reported Adverse Events from the Global ICSR Database VigiBase

From the analysis of the ICSRs in the global database VigiBase, it appeared that most AEs were non-serious. However, 15.8% were classified as serious, including eight fatalities. Of these fatal cases, six were associated with *Pelargonium sidoides*, marketed under trade names such as Umckaloabo^®^ and Pelagon. These products, primarily available in oral formulations like syrups and tablets, were used to treat respiratory tract infections or as general prophylaxis. In some cases, they were used off-label, including for indications such as pancreatic cancer.

One fatality was linked to subcutaneously injected *Viscum album* (marketed as Abnova Viscum A), administered as part of complementary cancer care. The patient, an elderly woman with biliary tract cancer, experienced gastrointestinal bleeding and abdominal pain. Although causality was assessed as “unlikely” by the physician, the case raises concerns about the parenteral administration of herbal products, particularly in vulnerable populations.

Another fatality involved *Silybum marianum* (Legalon), an oral product prescribed by a physician for inflammation. The report, submitted by a consumer, stated that the patient died while using the product, although the exact cause of death remained unclear. Causality assessments for all eight fatal cases were inconsistent: four lacked a classification, two were assessed as unlikely related, one as possibly related, and one as non-assessable. It is important to note that these assessments were made by the original reporters, pharmacovigilance centers, or marketing authorization holders (MAHs) who submitted the reports, and not by the UMC. Moreover, causality information is not consistently available for all cases.

### 3.2. Geographic and Population Trends of the ICSRs

The Western Pacific Region accounted for the highest proportion of reported AEs, comprising 44.7% of single-ingredient ICSRs and 85.4% of multi-ingredient ICSRs. This reflects the strong integration of herbal medicine in healthcare systems in countries such as China, Japan, South Korea, and Australia. Traditional Chinese Medicine (TCM), Kampo, and Hanbang medicine play a significant role in the widespread use of herbal products, increasing both population exposure and the potential for AEs. Among the single-ingredient products, *Ganoderma lucidum* was the most frequently reported, likely due to its longstanding use in TCM. Historical references date back to Shennong’s Herbal Classics (circa 25 AD), where it was highly esteemed for its medicinal properties [179].

The predominance of females was observed in the ICSRs, with women accounting for 58.2% compared to 39.1% for men. This aligns with well-established patterns in herbal medicine use, as women are generally more likely to consume herbal and dietary supplements, particularly those marketed for hormonal regulation, immune support, and stress adaptation [180]. Furthermore, it is recognized that women are more susceptible to experiencing AEs than men [181]. The most frequently reported age group was 45–64 years, which is consistent with the higher prevalence of herbal product use among older adults compared to younger individuals [180].

### 3.3. Multi-Ingredient Products

#### Adverse Events Reported in Clinical Trials from the Scoping Review

Across 54 clinical trials evaluating multi-ingredient natural products with claimed adaptogenic or immunomodulatory effects, no SAEs were reported. Overall, the safety profiles were favorable: 20 studies explicitly reported no AEs, and the remainder documented only mild to moderate reactions.

However, attributing AEs to a specific component within a multi-ingredient formulation remains inherently challenging. Given the complex composition of these products, current study designs do not allow for the establishment of a causal relationship between individual ingredients and observed AEs.

An interesting observation, however, is that nearly half of the case reports involving multi-ingredient products featured propolis, a resinous bee-derived substance frequently included in combination formulations. These cases were primarily associated with hypersensitivity and dermatological reactions, including contact dermatitis, systemic allergic responses, and anaphylaxis. This is particularly intriguing, as propolis is not a uniform substance but rather a complex mixture of plant resins collected by bees from various botanical sources. Its chemical composition can vary substantially depending on the geographic region, local flora, and even the season of collection. As a result, the allergenic potential of propolis will differ across formulations.

In the analysis of ICSRs, *Salvia miltiorrhiza* emerged as the most frequently reported natural product. This predominance likely reflects its extensive use in traditional and integrative medicine, particularly in the Western Pacific Region, where it is commonly prescribed for cardiovascular, cerebrovascular, and hepatic conditions.

### 3.4. Strengths and Limitations

The main strengths of this study lie in its comprehensive scoping review, which systematically evaluates the safety concerns and AEs associated with natural products possessing immunomodulatory and adaptogenic properties. By integrating findings from both clinical trials and case studies, the review provides a broad yet detailed overview of AEs reported in the scientific literature. Furthermore, the inclusion of pharmacovigilance data from the WHO-UMC global database VigiBase strengthens the analysis by capturing real-world safety signals, including rare but serious AEs that may not be detected in controlled clinical settings.

However, several limitations must be acknowledged. The literature search was restricted to PubMed, which may have resulted in missing relevant studies indexed in other databases, although PubMed remains one of the most comprehensive biomedical resources. It should also be noted that most natural products display a broad spectrum of biological activities beyond their adaptogenic and immunomodulatory effects. As such, some reported AEs may be attributable to other pharmacological actions of the product, particularly in individuals with co-morbidities or increased susceptibility or in the presence of confounding factors. We assumed that these natural products did not contain any registered pharmaceutical ingredients. However, the addition of illegal ingredients to natural products cannot always be ruled out. Additionally, underreporting is a well-recognized limitation in pharmacovigilance, as spontaneous reports rely on voluntary submissions. In addition, not all pharmacovigilance centers collect reports on unregistered herbal products, and some may not share these with international pharmacovigilance databases. This potentially leads to a distorted and incomplete picture of the true incidence and severity of AEs associated with these natural products.

Lastly, detailed case narratives were not available to the researchers, limiting the ability to conduct full causality assessments for individual cases.

## 4. Materials and Methods

### 4.1. Scoping Review

This scoping review was conducted to systematically map the existing literature on adaptogenic and immunomodulatory natural products, with a particular focus on their safety profiles and associated AEs [182]. The review followed a structured framework that is in line with the Preferred Reporting Items for Systematic Reviews and Meta-Analyses extension for Scoping Reviews (PRISMA-ScR) guidelines [183].

Eligibility criteria:

- Inclusion: Clinical trials, case reports, and randomized controlled trials (RCTs) involving human subjects that specifically assess the safety or AEs of herbal products and fungi with presumed adaptogenic or immunomodulatory properties.
- Exclusion: Review articles; studies solely based on animal or in vitro models; papers without accessible full texts or without mention of AEs; and publications in languages other than Dutch or English.

Search strategy:

The search was conducted in two stages: First, a broad exploratory search was performed using terms such as “plant adaptogens”, “plant immunomodulators”, “fungal adaptogens“, and “fungal immunomodulators” to identify specific natural products commonly referred to as “adaptogenic” or “immunomodulatory”. Based on this, a list of relevant species was compiled. Next, a more focused search was conducted for each identified species to gather information regarding their safety profiles and AEs. Using specific search terms that include the scientific names of the plants (e.g., *Withania somnifera*, *Panax ginseng*, *Echinacea purpurea*) and the common names, combined with terms related to safety, such as “safety”, “adverse reactions”, “adverse effects”, “toxicity”, and “side effects.” To ensure broader coverage, a more general search combining terms like “adaptogen” and “immunomodulatory” with “herbal products” and “fungi” was conducted. The search strategy is detailed in the Supplementary Materials (Table S8). Duplicate records were identified and removed using EndNote 21 [184].

### 4.2. Data Extraction and Analysis

#### 4.2.1. Scoping Review

Eligibility criteria were used to include or exclude the studies. Initially, titles and abstracts were screened, and in cases of uncertainty regarding eligibility, the full text was retrieved for further evaluation. Articles that did not meet the inclusion criteria upon full review were excluded. A PRISMA flow diagram was used to illustrate the study selection process. ASReview (version R 4.4.2) was utilized to assist with the screening of the articles [185]. The study protocol was pre-registered on the Open Science Framework (https://osf.io/), accessed on 28 October 2024 (registration DOI https://doi.org/10.17605/OSF.IO/C3SQN).

Data extraction was performed using a structured tool based on the Joanna Briggs Institute (JBI) template [186]. Extracted data were organized in a Microsoft Excel spreadsheet to maintain consistency and transparency. Key data fields included author, number of participants, study type, participant demographics (sex and age), study location, and objectives. Details on interventions were also recorded, including plant species (with Latin names), plant parts used, dosage, dosage form, any standardization information, dosage regimen, and comparator treatments, where applicable.

#### 4.2.2. Global Individual Case Safety Reports Database (WHO-UMC) VigiBase

To support the analysis of AE patterns related to adaptogenic and immunomodulatory natural products, structured tables were developed using data from ICSRs extracted from the WHO-UMC global pharmacovigilance database VigiBase through a targeted request. VigiBase, established by the Uppsala Monitoring Centre (UMC) in 1978, serves as the WHO’s global repository of suspected adverse drug reactions. The dataset included data from the inception of VigiBase until the extraction date (17 February 2025). The selection of natural products included in the search was based on the previous literature search as part of the scoping review. The selection of products searched in VigiBase was based on the list of 49 natural products identified during the literature search component of the scoping review. Key fields retrieved included case ID, report date, reporting country, and seriousness classification according to the Council for International Organizations of Medical Sciences (CIOMS criteria) [187], reporter qualification, patient demographics (age and sex), product name and ingredients, dosage, indication, time to onset, concomitant medications, reported AEs (classified by System Organ Class (SOC) and Preferred Term (PT)), and clinical outcomes.

The dataset was filtered to include only reports in which the natural product was designated as a suspect or interacting agent. A subset of this data was used to summarize the frequency with which each natural product appeared in the ICSRs, focusing on the 15 most frequently reported ingredients.

A second dataset was generated to explore associations between individual ingredients and AEs by focusing on the SOCs. For each ingredient, the top five most frequently reported SOCs were identified and presented. It is important to note that these figures represent only the total number of AEs reported within the top five SOCs and do not reflect the complete set of reactions for each ingredient. As such, the overall AE counts may be underestimated.

Further, for each SOC, the five most frequently reported PTs were listed, providing a detailed view of the most common clinical manifestations. However, as with the SOCs, only a partial picture of the total AE spectrum is presented. This structural approach enabled a more interpretable analysis of the AE patterns by highlighting both the most affected organ systems and the most common clinical manifestations associated with each natural product ingredient.

Due to data protection policies, the authors did not have access to the full narrative details of the reports. As a result, a full causality assessment of the reported AEs could not be conducted. When available, the causality classification assigned by the original reporting was used.

## 5. Conclusions

This scoping review and pharmacovigilance analysis show that natural products with claimed adaptogenic and immunomodulatory properties are widely used and generally perceived as safe. However, the broad heterogeneity of this group, including botanical, fungal, and animal-derived substances, leads to varied pharmacological actions and safety profiles. Therefore, generalizations are inappropriate; each species requires individual safety evaluation.

While many products were associated only with mild AEs, such as gastrointestinal discomfort, several serious and potentially life-threatening AEs were also documented. For example, *Viscum album*, administered subcutaneously in complementary cancer care, was associated with systemic hypersensitivity reactions. This route of administration falls outside the EMA definition of phytotherapy, highlighting both clinical and regulatory concerns.

Additional safety issues were noted with *Camellia sinensis*, linked to elevated liver enzymes, and *Glycyrrhiza glabra*, which caused severe arrhythmias through pseudoaldosteronism. These examples underscore that even commonly accepted “natural” products can present significant risks.

We recommend that health authorities consider explicit safety warnings for natural substances known to affect major organs or interact with conventional medications.

Due to unclear mechanisms, herb–drug interactions, and non-standardized dosing, caution is particularly warranted for individuals with pre-existing conditions or those concurrently taking conventional medicines. For instance, combining *Uncaria tomentosa* or *Bacopa monnieri* with serotonergic or cholinergic drugs may pose added risks and professional medical advice should be sought.

Given the growing use of adaptogens and immunomodulatory natural products, clearer product labeling and enhanced post-marketing surveillance are needed. Including known AEs and interaction risks in public and clinical guidelines can help promote safer use. Future research should prioritize robust clinical trials, standardized safety reporting, and stronger international phytovigilance to better inform both consumers and healthcare providers, ultimately helping to prevent unexpected AEs.

## Figures and Tables

**Table 1:** Overview of the number of studies on natural products with adaptogenic and/or immunomodulating properties.

Type of Study	Number of Studies with Single-Ingredient Products (n)	Number of Studies with Multi-Ingredient Products (n)
Non-randomized clinical trial	49	7
Randomized clinical trial	236	47
Case studies	119	21
Total	404	75

**Table 2:** Top ten natural products with the highest number of available clinical trials investigating adaptogenic and/or immunomodulating properties. Results include both single-ingredient and multi-ingredient products.

Natural Product	Number of Studies with Single-Ingredient Products (n)	Number of Studies with Multi-Ingredient Products (n)	Total (n) and % *
*Curcuma longa* L.	35	4	39 (11.5%)
*Panax ginseng* C.A. Meyer	24	1	25 (7.4%)
*Zingiber officinale* Roscoe	18	6	24 (7.1%)
*Withania somnifera* (L.) Dunal	18	4	22 (6.5%)
*Viscum album* L.	21	0	21 (6.2%)
*Silybum marianum* (L.) Gaertn.	16	5	21 (6.2%)
*Pelargonium sidoides* DC.	15	0	15 (4.4%)
*Echinacea purpurea* (L.) Moench	7	5	12 (3.5%)
*Aloe vera* (L.) Burm.f.	9	1	10 (2.9%)
*Rhodiola rosea* L.	8	1	9 (2.7%)

* Total of clinical studies on single-ingredient and multi-ingredient products n = 339.

**Table 3:** Top ten natural products with the highest number of case studies involving interventions with single-ingredient products claiming adaptogenic and/or immunomodulating properties.

Natural Product	Number of Case Studies (n) and % *
*Glycyrrhiza glabra* L.	15 (11.5%)
*Camellia sinensis* (L.) Kuntze	11 (8.5%)
*Lentinula edodes* (Berk.) Pegl.	11 (8.5%)
*Panax ginseng* C.A. Meyer	10 (7.7%)
*Allium sativum* L.	9 (6.9%)
*Aloe vera* (L.) Burm.f.	8 (6.2%)
*Curcuma longa* L.	8 (6.2%)
*Viscum album* L.	6 (4.6%)
*Azadirachta indica* A. Juss.	5 (3.8%)
*Morinda citrifolia* L.	5 (3.8%)

* Total number of case studies n = 119.

**Table 4:** Age and sex distribution of the ICSRs on single-ingredient and multiple-ingredient natural products.

Age Group	Number of ICSRs (n) and % *	Number of ICSRs of Single-Ingredient Products (n)	Number of ICSRs of Multi-Ingredient Products (n)
0–27 days	54 (0.1%)	25	29
28 days to 23 months	174 (0.4%)	97	77
2–11 years	607 (1.3%)	377	230
12–17 years	468 (1.0%)	214	254
18–44 years	7830 (17.4%)	2466	5364
45–64 years	15,922 (35.3%)	3537	12,385
65–74 years	8249 (18.3%)	1465	6784
≥75 years	6641 (14.7%)	903	5738
Unknown	5097 (11.3%)	1618	3479
**Sex**			
Female	26,233 (58.2%)	6354	19,879
Male	17,625 (39.1%)	4002	13,623
Unknown	1184 (2.6%)	346	838

* Calculated as the percentage of all reports (n = 45,042).

**Table 5:** Distribution of ICSRs of the single-ingredient and multi-ingredient natural products across WHO regions.

WHO Region	Number of ICSRs (n) and % of Single-Ingredient Products	Number of ICSRs (n) and % of Multi-Ingredient Products
Western Pacific Region	4786 (44.7%)	29,319 (85.4%)
European Region	3597 (33.6%)	3219 (9.4%)
Region of the Americas	1052 (9.8%)	951 (2.8%)
South-East Asia Region	1013 (9.5%)	707 (2.1%)
Eastern Mediterranean Region	220 (2.1%)	90 (0.3%)
African Region	34 (0.3%)	54 (0.2%)
Total	10,702	34,340

**Table 6:** Ten most frequently reported single-ingredient natural products identified as the sole suspect in ICSRs.

Natural Product	Number of Reports (n) and %
*Ganoderma lucidum* (Curtis) P. Karst	1529 (19.4%)
*Viscum album* L.	1358 (17.3%)
*Silybum marianum* (L.) Gaertn.	1103 (14.0%)
*Pelargonium sidoides* DC.	749 (9.5%)
*Andrographis paniculata* (Burm.f.) Wall. ex Nees	545 (6.9%)
*Rhodiola rosea* L.	484 (6.1%)
*Salvia miltiorrhiza* Bunge	472 (6.0%
*Curcuma longa* L.	318 (4.0%)
*Echinacea purpurea* (L.) Moench	295 (3.8%)
Propolis from *Apis mellifera*	126 (1.6%)

**Table 7:** The seriousness of the AEs and their impact, following the use of a single-ingredient natural product as the sole suspect of the AEs.

Seriousness of Reaction	Frequency (n) and % *	Impact of the Serious Reaction	Frequency (n)	Top Three Most Frequently Reported	Frequency (n) and % *
Serious	1242 (15.8%)	Caused/Prolonged Hospitalization	249	*Ganoderma lucidum*	435 (35.0%)
		Caused/Prolonged Hospitalization, Other	25	*Pelargonium sidoides*	171 (13.8%)
		Death	8	*Salvia miltiorrhiza*	133 (10.7%)
		Death, Caused/Prolonged Hospitalization, Disabling/Incapacitating	1		
		Disabling/Incapacitating	10		
		Disabling/Incapacitating, Other	5		
		Life-Threatening	35		
		Life-Threatening, Caused/Prolonged Hospitalization	25		
		Life-Threatening, Caused/Prolonged Hospitalization, Disabling/Incapacitating	1		
		Life-Threatening, Caused/Prolonged Hospitalization, Other	5		
		Life-Threatening, Other	3		
Not serious	5236 (66.6%)	Unknown	7209		
Unknown	1377 (17.5%)	Other	279		

* Calculated as the percentage of all reports where the single-ingredient product is the sole causative agent (n = 7856).

**Table 8:** Overview of the ten most frequently reported multi-ingredient natural products as the sole suspect involved in an ICSR.

Natural Product	Number of Reports (n)
*Salvia miltiorrhiza* Bunge	18,623
*Glycyrrhiza glabra* L.	1853
*Echinacea purpurea* (L.) Moench	1536
*Zingiber officinale* Roscoe	1378
*Panax ginseng* C.A. Meyer	1253
*Achyranthes bidentata* Blume	988
*Silybum marianum* (L.) Gaertn	980
*Andrographis paniculata* (Burm.f.) Wall. ex Nees	836
*Terminalia chebula* Retz.	528
*Allium sativum* L.	504

## Data Availability

The datasets for this manuscript are not publicly available because of the Lareb data protection policy. Requests to access the dataset should be directed to the last author (F.P.A.M.v.H.) and will be granted on reasonable request.

## Supplementary Materials

The following supporting information can be downloaded at https://www.mdpi.com/article/10.3390/ph18081208/s1, Table S1. Overview of clinical studies on single-herb and multi-herb products from the scoping review, arranged by natural product; Table S2. Overview of case studies on single-herb products from the scoping review, arranged by natural product; Table S3. Overview of case studies on single-herb products from the scoping review, arranged by natural product; Table S4. Overview of the top 15 most reported single-ingredient natural products for all reported adverse events, with the SOCs and their respective PTs; Table S5. Overview of the top 15 most reported single-ingredient natural products for the serious adverse events, with the SOCs and their respective PTs; Table S6. Overview of the reported multi-ingredient natural products involved in an ICSR as sole suspect; Table S7. Overview of the top 15 most reported multi-ingredient natural products with the SOCs and their respective PTs; Table S8. Search strategy applied to the scoping review in PubMed.

## Abbreviations

The following abbreviations are used in this manuscript:

AE(s)	Adverse Event(s)
ALT	Alanine Aminotransferase
AST	Aspartate Aminotransferase
EMA	European Medicines Agency
EGCG	Epigallocatechin Gallate
FDA	Food and Drug Administration
GTE	Green Tea Extract
ICSR(s)	Individual Case Safety Report(s)
JBI	Joanna Briggs Institute
MAH(s)	Marketing Authorization Holder(s)
MRI	Magnetic Resonance Imaging
PRISMA-ScR	Preferred Reporting Items for Systematic Reviews and Meta-Analyses Extension for Scoping Reviews
PT(s)	Preferred Term(s)
RCT(s)	Randomized Controlled Trial(s)
RUCAM	Roussel Uclaf Causality Assessment Method
SAE(s)	Serious Adverse Event(s)
SOC(s)	System Organ Class(es)
TCM	Traditional Chinese Medicine
UMC	Uppsala Monitoring Centre
WHO	World Health Organization
