# Supplementary material for: Safety Considerations for Natural Products with Adaptogenic and Immunomodulating Activities
Source: Pharmaceuticals (Basel). 2025 Aug 15;18(8):1208. doi: 10.3390/ph18081208 (PMC12389708; doi:10.3390/ph18081208)
Supplement: Supplementary file 1 [file pharmaceuticals-18-01208-s001.zip › pharmaceuticals-3745734-supplementary.pdf]

*Supplementary Materials*

# Safety Considerations for Natural Products with Adaptogenic and Immunomodulating Activities

*Chen Jia Wen Liang[1], Herman J. Woerdenbag[2], Corine Ekhart[3], Annabella Vitalone[4] and Florence P. A. M. van Hunsel[3,5,*]*

1 Pharmacy Master Programme, School of Science and Engineering, University of Groningen, Antonius Deusinglaan 1, 9713 AV Groningen, The Netherlands; c.j.w.liang@student.rug.nl (C.J.W.L.)

2 Department of Pharmaceutical Technology and Biopharmacy, Groningen Research Institute of Pharmacy (GRIP), University of Groningen, Antonius Deusinglaan 1, 9713 AV Groningen, The Netherlands; h.j.woerdenbag@rug.nl (H.J.W.)

3 Netherlands Pharmacovigilance Centre Lareb, Goudsbloemvallei 7, 5237 MH 's-Hertogenbosch, The Netherlands; c.ekhart@lareb.nl (C.E.)

4 Department of Physiology and Pharmacology 'Vittorio Erspamer', Sapienza University of Rome, Piazzale Aldo Moro 5, 00185 Rome, Italy; annabella.vitalone@uniroma1.it (A.V.)

5 Department of PharmacoTherapy, -Epidemiology & -Economics, Groningen Research Institute of Pharmacy (GRIP), University of Groningen, Antonius Deusinglaan 1, 9713 AV Groningen, The Netherlands

* Correspondence: f.vanhunsel@lareb.nl

**Table of Contents**

**Table S1**. Overview of clinical studies on single-herb and multi-herb products from the scoping review, arranged by natural product.

**Table S2**. Overview of case studies on single-herb products from the scoping review, arranged by natural product.

**Table S3**. Overview of case studies on single-herb products from the scoping review, arranged by natural product.

**Table S4**. Overview of the top 15 most reported single-ingredient natural products for all reported adverse events, with the SOCs and their respective PTs.

**Table S5**. Overview of the top 15 most reported single-ingredient natural products for the serious adverse events, with the SOCs and their respective PTs.

**Table S6**. Overview of the reported multi-ingredient natural products involved in an ICSR as the sole suspect.

**Table S7.** Overview of the top 15 most reported multi-ingredient natural products, with the SOCs and their respective PTs.

**Table S8**. Search strategy applied to the scoping review in PubMed.

**Table S1**. Overview of clinical studies on single-herb and multi-herb products from the scoping review, arranged by natural product.

| Natural product | Number of single-herb clinical studies (n) | Number of multi-herb clinical studies (n) | Total (n) and % |
|---|---|---|---|
| *Curcuma longa* L. | 35 | 4 | 39 (11.5%) |
| *Panax ginseng* C.A. Meyer | 24 | 1 | 25 (7.4%) |
| *Zingiber officinale* Roscoe | 18 | 6 | 24 (7.1%) |
| *Withania somnifera* (L.) Dunal | 18 | 4 | 22 (6.5%) |
| *Viscum album* L. | 21 | 0 | 21 (6.2%) |
| *Silybum marianum* (L.) Gaertn. | 16 | 5 | 21 (6.2%) |
| *Pelargonium sidoides* DC. | 15 | 0 | 15 (4.4%) |
| *Echinacea purpurea* (L.) Moench | 7 | 5 | 12 (3.5%) |
| *Aloe vera (L.)* Burm.f. | 9 | 1 | 11 (3.2%) |
| *Rhodiola rosea* L. | 8 | 1 | 9 (2.7%) |
| *Artemisia annua* L. | 8 | 0 | 8 (2.4%) |
| *Propolis* from *Apis mellifera* | 6 | 2 | 8 (2.4%) |
| *Allium sativum* L. | 6 | 1 | 7 (2.1%) |
| *Glycyrrhiza glabra* L. | 4 | 3 | 7 (2.1%) |
| *Panax quinquefolius* L. | 6 | 1 | 7 (2.1%) |
| *Phyllanthus emblica* L. | 4 | 3 | 7 (2.1%) |
| *Salvia officinalis* L. | 6 | 1 | 7 (2.1%) |
| *Astragalus membranaceus* Moench | 4 | 2 | 6 (1.8%) |
| *Ganoderma lucidum* (Curtis) P. Karst | 5 | 1 | 6 (1.8%) |
| *Camellia sinensis* (L.) Kuntze | 5 | 0 | 5 (1.5%) |
| *Eleutherococcus senticosus* (Rupr. & Maxim.) Maxim. | 2 | 3 | 5 (1.5%) |
| *Gynostemma pentaphyllum* (Thunb.) Makino | 4 | 1 | 5 (1.5%) |
| *Lycium barbarum* L. | 5 | 0 | 5 (1.5%) |
| *Lentinula edodes* (Berk.) Pegl. | 4 | 0 | 4 (1.2%) |
| *Momordica charantia* L. | 4 | 0 | 4 (1.2%) |
| *Terminalia chebula* Retz. | 2 | 2 | 4 (1.2%) |
| *Antrodia cinnamomea* | 3 | 0 | 3 (0.9%) |
| *Azadirachta indica* A.Juss. | 2 | 1 | 3 (0.9%) |
| *Bacopa monnieri* (L.) Wettst. | 3 | 0 | 3 (0.9%) |

| | | | |
|---|---|---|---|
| *Schisandra chinensis* (Turcz.) Baill. | 2 | 1 | 3 (0.9%) |
| *Grifola frondosa* (Dicks.) Gray | 2 | 0 | 2 (0.6%) |
| *Morinda citrifolia* L. | 2 | 0 | 2 (0.6%) |
| *Uncaria guianensis* (Aubl.) J.F.Gmel. | 1 | 1 | 2 (0.6%) |
| *Agaricus blazei Murrill* (ABM) | 1 | 0 | 1 (0.3%) |
| *Andrographis paniculata* (Burm.f.) Wall. ex Nees | 9 | 2 | 1 (0.3%) |
| *Aronia melanocarpa* (Michx.) Elliott | 1 | 0 | 1 (0.3%) |
| *Berberis vulgaris* L. | 1 | 0 | 1 (0.3%) |
| *Carica papaya* L. | 1 | 0 | 1 (0.3%) |
| *Cordyceps cicadae* | 1 | 0 | 1 (0.3%) |
| *Cordyceps militaris* (L.) Fr. | 1 | 0 | 1 (0.3%) |
| *Echinacea angustifolia* | 1 | 0 | 1 (0.3%) |
| *Hericium erinaceus* (Bull.) Pers. | 1 | 0 | 1 (0.3%) |
| *Ophiocordyceps sinensis* (Berk.) Sacc. | 1 | 0 | 1 (0.3%) |
| *Panax notoginseng* (Burkill) F.H. Chen | 1 | 0 | 1 (0.3%) |
| *Pleurotus ostreatus* (Jacq.Fr.) P. Kumm. | 1 | 0 | 1 (0.3%) |
| *Uncaria tomentosa* (Willd. ex Schult.) DC. | 1 | 0 | 1 (0.3%) |

**Table S2**. Overview of case studies on single-herb products from the scoping review, arranged by natural product.

| Natural Product | Number of Case Studies (n) and % |
|---|---|
| *Glycyrrhiza glabra* L. | 15 (11.5%) |
| *Camellia sinensis* (L.) Kuntze | 11 (8.5%) |
| *Lentinula edodes* (Berk.) Pegl. | 11 (8.5%) |
| *Panax ginseng* C.A. Meyer | 10 (7.7%) |
| *Allium sativum* L. | 9 (6.9%) |
| *Aloe vera*(L.) Burm.f. | 8 (6.2%) |
| *Curcuma longa* L. | 8 (6.2%) |
| *Viscum album* L. | 6 (4.6%) |
| *Azadirachta indica* A. Juss. | 5 (3.8%) |
| *Morinda citrifolia* L. | 5 (3.8%) |
| *Echinacea purpurea* (L.) Moench | 3 (2.3%) |
| *Lycium barbarum* L. | 3 (2.3%) |
| *Uncaria tomentosa* (Willd. ex Schult.) DC. | 3 (2.3%) |
| *Bacopa monnieri* (L.) Wettst. | 2 (1.5%) |
| *Eleutherococcus senticosus (Rupr. & Maxim.) Maxim.* | 2 (1.5%) |
| *Ganoderma lucidum* (Curtis) P. Karst | 2 (1.5%) |
| *Inonotus obliquus* (Ach. ex Pers.) Pilat. | 2 (1.5%) |
| *Tinospora crispa* (L.) Hook.f. & Thomson | 2 (1.5%) |
| *Achyranthes bidentata* Blume | 1 (0.8%) |
| *Agaricus blazei* Murrill (ABM) | 1 (0.8%) |
| *Artemisia annua* L. | 1 (0.8%) |
| *Astragalus membranaceus* Moench | 1 (0.8%) |
| *Carica papaya* L. | 1 (0.8%) |
| *Eriobotrya japonica (Thunb.) Lindl.* | 1 (0.8%) |
| *Momordica charantia* L. | 1 (0.8%) |
| *Ophiocordyceps sinensis* (Berk.) Sacc. | 1 (0.8%) |
| *Panax notoginseng (Burkill) F.H. Chen* | 1 (0.8%) |
| *Pelargonium sidoides* DC. | 1 (0.8%) |
| *Pleurotus ostreatus* (Jacq.Fr.) P. Kumm. | 1 (0.8%) |
| *Salvia officinalis* L. | 1 (0.8%) |
| *Silybum marianum* (L.) Gaertn. | 1 (0.8%) |
| Total | 130 |

**Table S3**. Overview of case studies on single-herb products from the scoping review, arranged by natural product.

| Natural Product | Number of Reports (n) and % |
|---|---|
| *Ganoderma lucidum* (Curtis) P. Karst | 1529 (19.4%) |
| *Viscum album* L. | 1358 (17.3%) |
| *Silybum marianum* (L.) Gaertn. | 1103 (14.0%) |
| *Pelargonium sidoides* DC. | 749 (9.5%) |
| *Andrographis paniculata* (Burm.f.) Wall. ex Nees | 545 (6.9%) |
| *Rhodiola rosea* L. | 484 (6.1%) |
| *Salvia miltiorrhiza* Bunge | 472 (6.0% |
| *Curcuma longa* L. | 318 (4.0%) |
| *Echinacea purpurea* (L.) Moench | 296 (3.8%) |
| Propolis from *Apis mellifera* | 126 (1.6%) |
| *Zingiber officinale* Roscoe | 111 (1.4%) |
| *Panax ginseng* C.A. Meyer | 99 (1.3%) |
| *Salvia officinalis* L. | 94 (1.2%) |
| *Allium sativum* L. | 90 (1.1%) |
| *Camellia sinensis* (L.) Kuntze | 78 (1.0%) |
| *Aloe vera* (L.) Burm.*f.* | 73 (0.9%) |
| *Panax quinquefolius* L. | 50 (0.6%) |
| *Glycyrrhiza glabra* L. | 46 (0.6%) |
| *Withania somnifera* (L.) Dunal | 39 (0.5%) |
| *Morinda citrifolia* L. | 33 (0.4%) |
| *Carica papaya* L. | 31 (0.4%) |
| *Astragalus membranaceus* Moench | 29 (0.4%) |
| *Eleutherococcus senticosus* (Rupr. & Maxim.) Maxim. | 20 (0.3%) |
| *Schisandra chinensis* (Turcz.) Baill. | 16 (0.2%) |
| *Lepidium meyenii* Walp | 13 (0.2%) |
| *Azadirachta indica* A. Juss. | 12 (0.1%) |
| *Artemisia annua* L. | 9 (0.1%) |
| *Momordica charantia* L. | 7 (0.09%) |
| *Panax notoginseng* **(**Burkill) F.H. Chen | 7 (0.09%) |
| *Bacopa monnieri* (L.) Wettst. | 6 (0.08%) |
| *Uncaria tomentosa* (Willd. ex Schult.) DC. | 4 (0.05%) |
| *Hericium erinaceus* (Bull.) Pers. | 3 (0.04%) |
| *Lentinula edodes* (Berk.) Pegl. | 3 (0.04%) |
| *Lycium barbarum* L. | 3 (0.04%) |
| *Ophiocordyceps sinensis* (Berk.) Sacc. | 3 (0.04%) |
| *Berberis vulgaris* L. | 2 (0.03%) |
| *Phyllanthus emblica* L. | 2 (0.03%) |
| *Tinospora crispa* (L.) Hook.f. & Thomson | 2 (0.03%) |
| *Cordyceps militaris* (L.) Fr. | 1 (0.01%) |
| Total | 7866 |

**Table S4**. Overview of the top 15 most reported single-ingredient natural products for all reported adverse events, with the SOCs and their respective PTs.

*Ganoderma lucidum* (Curtis) P. Karst

| System Organ Class (SOC) | Frequency (n) | Preferred term (PT) | Frequency (n) |
|---|---|---|---|
| **General disorders and administration site conditions** | 846 | Chills | 391 |
| | | Chest pain | 197 |
| | | Pyrexia | 164 |
| | | Hyperpyrexia | 94 |
| **Skin and subcutaneous tissue disorders** | 363 | Pruritus | 179 |
| | | Rash | 123 |
| | | Erythema | 31 |
| | | Hyperhidrosis | 30 |
| **Gastrointestinal disorders** | 360 | Nausea | 192 |
| | | Vomiting | 119 |
| | | Abdominal pain | 36 |
| | | Abdominal comfort | 13 |
| **Nervous system disorders** | 256 | Dizziness | 136 |
| | | Headache | 53 |
| | | Tremor | 34 |
| | | Hypoaestesia | 33 |
| **Cardiac disorders** | 212 | Palpitations | 128 |
| | | Cardiac flutter | 67 |
| | | Cardiac discomfort | 10 |
| | | Arrhythmia | 7 |

*Viscum album* L.

| System Organ Class (SOC) | Frequency (n) | Preferred term (PT) | Frequency (n) |
|---|---|---|---|
| **General disorders and administration site conditions** | 496 | Injection site inflammation | 284 |
| | | Pyrexia | 92 |
| | | Influenza like illness | 60 |
| | | Chills | 50 |
| **Skin and subcutaneous tissue disorders** | 437 | Pruritus | 164 |
| | | Rash | 106 |
| | | Urticaria | 94 |
| | | Erythema | 73 |
| **Gastrointestinal disorders** | 130 | Nausea | 59 |
| | | Diarrhoea | 28 |
| | | Vomiting | 24 |
| | | Abdominal pain | 19 |
| **Nervous system disorders** | 116 | Headache | 53 |
| | | Dizziness | 44 |
| | | Burning sensation | 10 |
| | | Somnolence | 9 |

| | | | |
|---|---|---|---|
| **Respiratory, thoracic and mediastinal disorders** | 67 | Dyspnoea | 40 |
| | | Bronchospasm | 6 |
| | | Cough | 6 |
| | | Asthma | 5 |

*Silybum marianum* (L.) Gaertn.

| System Organ Class (SOC) | Frequency (n) | Preferred term (PT) | Frequency (n) |
|---|---|---|---|
| **Gastrointestinal disorders** | 531 | Diarrhoea | 183 |
| | | Abdominal discomfort | 147 |
| | | Nausea | 142 |
| | | Abdominal pain | 59 |
| **Skin and subcutaneous tissue disorders** | 293 | Pruritus | 121 |
| | | Rash | 89 |
| | | Urticaria | 67 |
| | | Hyperhidrosis | 16 |
| **Nervous system disorders** | 96 | Dizziness | 48 |
| | | Headache | 31 |
| | | Somnolence | 11 |
| | | Dysgeusia | 6 |
| **Respiratory, thoracic and mediastinal disorders** | 45 | Dyspnoea | 28 |
| | | Cough | 4 |
| | | Epistaxis | 4 |
| | | Dysphonia | 3 |
| **General disorders and administration site conditions** | 43 | Asthenia | 11 |
| | | Pyrexia | 9 |
| | | Malaise | 9 |
| | | Face oedema | 7 |

*Pelargonium sidoides* DC.

| System Organ Class (SOC) | Frequency (n) | Preferred term (PT) | Frequency (n) |
|---|---|---|---|
| **Skin and subcutaneous tissue disorders** | 228 | Rash | 82 |
| | | Pruritus | 60 |
| | | Urticaria | 56 |
| | | Erythema | 30 |
| **Gastrointestinal disorders** | 213 | Diarrhoea | 74 |
| | | Nausea | 55 |
| | | Vomiting | 49 |
| | | Dyspepsia | 35 |
| **Respiratory, thoracic and mediastinal disorders** | 111 | Epistaxis | 65 |
| | | Dyspnoea | 25 |
| | | Cough | 11 |
| | | Haemoptysis | 5 |
| **Nervous system disorders** | 64 | Dizziness | 26 |
| | | Headache | 20 |
| | | Somnolence | 11 |
| | | Dysgeusia | 7 |

| | | | |
|---|---|---|---|
| **Immune system disorders** | 44 | Hypersensitivity | 41 |
| | | Immune system disorder | 1 |
| | | Anaphylactic reaction | 1 |
| | | Anaphylactic shock | 1 |

*Andrographis paniculata* (Burm.f.) Wall. ex Nees

| System Organ Class (SOC) | Frequency (n) | Preferred term (PT) | Frequency (n) |
|---|---|---|---|
| **Skin and subcutaneous tissue disorders** | 327 | Urticaria | 98 |
| | | Rash | 86 |
| | | Rash maculo-papular | 83 |
| | | Angioedema | 60 |
| **Immune system disorders** | 77 | Anaphylactic reaction | 51 |
| | | Anaphylactic shock | 20 |
| | | Hypersensitivity | 4 |
| | | Allergic oedema | 1 |
| **Gastrointestinal disorders** | 46 | Nausea | 17 |
| | | Vomiting | 13 |
| | | Lip swelling | 8 |
| | | Oedema mouth | 8 |
| **General disorders and administration site conitions** | 41 | Face oedema | 15 |
| | | Chest discomfort | 12 |
| | | Chest pain | 7 |
| | | Fatigue | 7 |
| **Respiratory, thoracic and mediastinal disorders** | 32 | Dyspnoea | 18 |
| | | Cough | 5 |
| | | Bronchospasm | 2 |
| | | Respiratory distress | 1 |

*Rhodiola rosea* L.

| System Organ Class (SOC) | Frequency (n) | Preferred term (PT) | Frequency (n) |
|---|---|---|---|
| **Nervous system disorders** | 166 | Headache | 72 |
| | | Dizziness | 42 |
| | | Somnolence | 42 |
| | | Tremor | 10 |
| **Psychiatric disorders** | 102 | Insomnia | 50 |
| | | Agitation | 21 |
| | | Restlessness | 17 |
| | | Anxiety | 14 |
| **Gastrointestinal disorders** | 99 | Nausea | 59 |
| | | Diarrhoea | 15 |
| | | Abdominal pain upper | 14 |
| | | Vomiting | 11 |
| **Cardiac disorders** | 60 | Tachycardia | 33 |
| | | Palpitations | 20 |
| | | Arrhythmia | 5 |
| | | Cardiovascular disorder | 2 |

| | | | |
|---|---|---|---|
| **Injury, poisoning and procedural complications** | 59 | Off label use | 26 |
| | | Underdose | 18 |
| | | Intentional underdose | 8 |
| | | Intentional product use issue | 7 |

*Salvia miltiorrhiza* Bunge

| System Organ Class (SOC) | Frequency (n) | Preferred term (PT) | Frequency (n) |
|---|---|---|---|
| **Gastrointestinal disorders** | 167 | Nausea | 63 |
| | | Diarrhoea | 45 |
| | | Vomiting | 30 |
| | | Abdominal pain | 29 |
| **Skin and subcutaneous tissue disorders** | 161 | Pruritus | 73 |
| | | Rash | 57 |
| | | Erythema | 17 |
| | | Drug eruption | 14 |
| **General disorders and administration site conditions** | 80 | Chest pain | 43 |
| | | Chills | 19 |
| | | Pyrexia | 10 |
| | | Hyperpyrexia | 8 |
| **Nervous system disorders** | 50 | Dizziness | 34 |
| | | Headache | 11 |
| | | Hypoaesthesia | 3 |
| | | Head discomfort | 2 |
| **Respiratory, thoracic and mediastinal disorders** | 48 | Dyspnoea | 31 |
| | | Tachypnoea | 7 |
| | | Cough | 6 |
| | | Suffocation feeling | 4 |

*Curcuma longa* L.

| System Organ Class (SOC) | Frequency (n) | Preferred term (PT) | Frequency (n) |
|---|---|---|---|
| **Gastrointestinal disorders** | 155 | Abdominal pain | 50 |
| | | Nausea | 43 |
| | | Diarrhoea | 38 |
| | | Eructation | 24 |
| **General disorders and administration site conditions** | 148 | Fatigue | 60 |
| | | Chest discomfort | 46 |
| | | Therapeutic response decreased | 3 |
| | | Pyrexia | 9 |
| **Skin and subcutaneous tissue disorders** | 62 | Pruritus | 27 |
| | | Rash | 21 |
| | | Urticaria | 7 |
| | | Rash maculo-papular | 7 |
| **Nervous system disorders** | 61 | Headache | 46 |
| | | Dizziness | 5 |
| | | Somnolence | 3 |
| | | Nervous system disorder | 1 |

| | | | |
|---|---|---|---|
| **Respiratory, thoracic and mediastinal disorders** | 52 | Dyspnoea | 34 |
| | | Cough | 17 |
| | | Bronchospasm | 1 |

*Echinacea purpurea* (L.) Moench

| System Organ Class (SOC) | Frequency (n) | Preferred term (PT) | Frequency (n) |
|---|---|---|---|
| **Skin and subcutaneous tissue disorders** | 91 | Rash | 30 |
| | | Urticaria | 25 |
| | | Pruritus | 23 |
| | | Rash erythematous | 13 |
| **Gastrointestinal disorders** | 69 | Nausea | 24 |
| | | Diarrhoea | 20 |
| | | Abdominal pain | 14 |
| | | Vomiting | 11 |
| **Nervous system disorders** | 39 | Headache | 10 |
| | | Dizziness | 10 |
| | | Dysgeusia | 5 |
| | | Somnolence | 2 |
| **Respiratory, thoracic and mediastinal disorders** | 32 | Dyspnoea | 18 |
| | | Cough | 8 |
| | | Throat tightness | 3 |
| | | Throat irritation | 3 |
| **General disorders and administration site conditions** | 24 | Pyrexia | 7 |
| | | Malaise | 6 |
| | | Face oedema | 6 |
| | | Chills | 5 |

Propolis from *Apis mellifera*

| System Organ Class (SOC) | Frequency (n) | Preferred term (PT) | Frequency (n) |
|---|---|---|---|
| **Skin and subcutaneous tissue disorders** | 51 | Rash | 20 |
| | | Pruritus | 17 |
| | | Urticaria | 7 |
| | | Erythema | 6 |
| **Gastrointestinal disorders** | 34 | Vomiting | 14 |
| | | Nausea | 14 |
| | | Abdominal pain | 3 |
| | | Diarrhoea | 3 |
| **Unknown** | 16 | - | - |
| **General disorders and administration site conditions** | 15 | Face oedema | 6 |
| | | Drug ineffective | 3 |
| | | Malaise | 2 |
| | | Swelling face | 2 |
| **Respiratory, thoracic and mediastinal disorders** | 14 | Dyspnoea | 7 |
| | | Bronchospasm | 4 |
| | | Throat irritation | 2 |
| | | Pharyngeal oedema | 1 |

*Zingiber officinale* Roscoe

| System Organ Class (SOC) | Frequency (n) | Preferred term (PT) | Frequency (n) |
|---|---|---|---|
| **Gastrointestinal disorders** | 40 | Vomiting | 15 |
| | | Nausea | 10 |
| | | Abdominal pain | 8 |
| | | Diarrhoea | 7 |
| **Skin and subcutaneous tissue disorders** | 36 | Rash | 14 |
| | | Urticaria | 9 |
| | | Pruritus | 7 |
| | | Erythema | 3 |
| **Nervous system disorders** | 23 | Headache | 7 |
| | | Somnolence | 5 |
| | | Dizziness | 3 |
| | | Tremor | 2 |
| **General disorders and administration site conditions** | 19 | Drug ineffective | 4 |
| | | Unevaluable event | 2 |
| | | Asthenia | 1 |
| | | Swelling face | 1 |
| **Renal and urinary disorders** | 11 | Haematuria | 2 |
| | | Bladder irritation | 1 |
| | | Polyuria | 1 |
| | | Pollakiuria | 1 |

*Panax ginseng* C.A. Meyer

| System Organ Class (SOC) | Frequency (n) | Preferred term (PT) | Frequency (n) |
|---|---|---|---|
| **Gastrointestinal disorders** | 36 | Diarrhoea | 12 |
| | | Nausea | 10 |
| | | Abdominal pain upper | 5 |
| | | Abdominal pain | 4 |
| **Nervous system disorders** | 25 | Dizziness | 14 |
| | | Headache | 7 |
| | | Tremor | 2 |
| | | Paraesthesia | 2 |
| **Psychiatric disorders** | 18 | Anxiety | 3 |
| | | Nervousness | 3 |
| | | Insomnia | 3 |
| | | Restlessness | 1 |
| **General disorders and administration site conditions** | 17 | Fatigue | 3 |
| | | Feeling hot | 3 |
| | | Malaise | 2 |
| | | Oedema peripheral | 1 |
| **Skin and subcutaneous tissue disorders** | 14 | Rash | 5 |
| | | Urticaria | 3 |
| | | Erythema | 3 |
| | | Pruritus | 3 |

*Salvia officinalis* L.

| System Organ Class (SOC) | Frequency (n) | Preferred term (PT) | Frequency (n) |
|---|---|---|---|
| **Gastrointestinal disorders** | 38 | Vomiting | 11 |
| | | Nausea | 10 |
| | | Abdominal pain | 8 |
| | | Diarrhoea | 8 |
| **Skin and subcutaneous tissue disorders** | 22 | Pruritus | 6 |
| | | Erythema | 4 |
| | | Rash | 3 |
| | | Urticaria | 3 |
| **Nervous system disorders** | 18 | Dizziness | 4 |
| | | Migraine | 2 |
| | | Seizure | 2 |
| | | Paraesthesia | 2 |
| **General disorders and administration site conditions** | 16 | Pain | 4 |
| | | Asthenia | 3 |
| | | Drug ineffective | 3 |
| | | Fatigue | 2 |
| **Injury, poisoning and procedural complications** | 11 | Overdose | 3 |
| | | Incorrect route of product administration | 3 |
| | | Medication error | 2 |
| | | Wrong product administered | 1 |

*Allium sativum* L.

| System Organ Class (SOC) | Frequency (n) | Preferred term (PT) | Frequency (n) |
|---|---|---|---|
| **Gastrointestinal disorders** | 27 | Dyspepsia | 9 |
| | | Abdominal pain upper | 8 |
| | | Nausea | 5 |
| | | Diarrhoea | 5 |
| **Skin and subcutaneous tissue disorders** | 17 | Pruritus | 6 |
| | | Rash | 5 |
| | | Hyperhidrosis | 2 |
| | | Erythema | 2 |
| **General disorders and administration site conditions** | 16 | Pain | 4 |
| | | Peripheral swelling | 3 |
| | | Malaise | 3 |
| | | Asthenia | 2 |
| **Nervous system disorders** | 14 | Dizziness | 5 |
| | | Hypoaesthesia | 4 |
| | | Headache | 3 |
| | | Burning sensation | 2 |
| **Vascular disorders** | 8 | Hypotension | 3 |
| | | Vascular pain | 1 |
| | | Peripheral coldness | 1 |
| | | Flushing | 1 |

*Camellia sinensis* (L.) Kuntze

| System Organ Class (SOC) | Frequency (n) | Preferred term (PT) | Frequency (n) |
|---|---|---|---|
| **Gastrointestinal disorders** | 28 | Nausea | 8 |
| | | Vomiting | 6 |
| | | Abdominal pain | 5 |
| | | Abdominal pain upper | 3 |
| **Hepatobiliary disorders** | 23 | Hepatitis | 10 |
| | | Jaundice | 5 |
| | | Hepatitis cholestatic | 4 |
| | | Hepatitis acute | 4 |
| **General disorders and administration site conditions** | 13 | Fatigue | 4 |
| | | Asthenia | 2 |
| | | Oedema | 2 |
| | | Malaise | 1 |
| **Psychiatric disorders** | 13 | Insomnia | 3 |
| | | Agitation | 2 |
| | | Nervousness | 2 |
| | | Anxiety | 1 |
| **Investigations** | 13 | Tri-iodothyronine increased | 2 |
| | | Liver function test abnormal | 2 |
| | | Blood glucose increased | 2 |
| | | Myoglobin blood present | 1 |

**Table S5**. Overview of the top 15 most reported single-ingredient natural products for the serious adverse events; along with the SOCs and the respective PTs.

*Ganoderma lucidum (Curtis*) P. Karst

| System Organ Class (SOC) | Frequency (n) | Preferred term (PT) | Frequency (n) |
|---|---|---|---|
| **General disorders and administration site conditions** | 415 | Chills | 194 |
| | | Hyperpyrexia | 83 |
| | | Pyrexia | 81 |
| | | Chest pain | 57 |
| **Skin and subcutaneous tissue disorders** | 105 | Pruritus | 42 |
| | | Rash | 42 |
| | | Hyperhidrosis | 12 |
| | | Erythema | 9 |
| **Respiratory, thoracic and mediastinal disorders** | 87 | Dyspnoea | 59 |
| | | Tachypnoea | 15 |
| | | Wheezing | 5 |
| | | Cough | 4 |
| **Gastrointestinal disorders** | 60 | Nausea | 25 |
| | | Vomiting | 24 |
| | | Abdominal pain | 3 |
| | | Diarrhoea | 1 |
| **Immune system disorders** | 60 | Anaphylactoid reaction | 28 |
| | | Anaphylactic shock | 27 |
| | | Anaphylactic reaction | 4 |
| | | Hypersensitivity | 1 |

*Pelargonium sidoides* DC.

| System Organ Class (SOC) | Frequency (n) | Preferred term (PT) | Frequency (n) |
|---|---|---|---|
| **Skin and subcutaneous tissue disorders** | 55 | Rash | 20 |
| | | Pruritus | 17 |
| | | Erythema | 9 |
| | | Urticaria | 9 |
| **Respiratory, thoracic and mediastinal disorders** | 35 | Dyspnoea | 15 |
| | | Epistaxis | 14 |
| | | Pharyngeal swelling | 3 |
| | | Haemoptysis | 3 |
| **Hepatobiliary disorders** | 32 | Jaundice | 11 |
| | | Drug-induced liver injury | 7 |
| | | Hepatitis | 5 |
| | | Liver disorder | 3 |

| | | | |
|---|---|---|---|
| **Investigations** | 24 | Transaminases increased | **7** |
| | | Aspartate aminotransferase increased | 3 |
| | | Gamma-glutamyltransferase increased | 2 |
| | | Alanine aminotransferase abnormal | 2 |
| **Gastrointestinal disorders** | 19 | Abdominal pain upper | 5 |
| | | Nausea | 5 |
| | | Diarrhoea | 5 |
| | | Vomiting | 4 |

*Salvia miltiorrhiza* Bunge

| System Organ Class (SOC) | Frequency (n) | Preferred term (PT) | Frequency (n) |
|---|---|---|---|
| **Skin and subcutaneous tissue disorders** | 73 | Pruritus | 29 |
| | | Rash | 22 |
| | | Drug eruption | 13 |
| | | Erythema | 8 |
| **General disorders and administration site conditions** | 68 | Chest pain | 34 |
| | | Chills | 18 |
| | | Pyrexia | 8 |
| | | Hyperpyrexia | 8 |
| **Respiratory, thoracic and mediastinal disorders** | 43 | Dyspnoea | 28 |
| | | Tachypnoea | 7 |
| | | Suffocation feeling | 4 |
| | | Cough | 4 |
| **Vascular disorders** | 19 | Flushing | 10 |
| | | Cyanosis | 5 |
| | | Pallor | 2 |
| | | Peripheral coldness | 1 |
| **Immune system disorders** | 15 | Anaphylactic shock | 6 |
| | | Anaphylactic reaction | 5 |
| | | Anaphylactoid reaction | 4 |

*Andrographis paniculata* (Burm.f.) Wall. ex Nees

| System Organ Class (SOC) | Frequency (n) | Preferred term (PT) | Frequency (n) |
|---|---|---|---|
| **Immune system disorders** | 38 | Anaphylactic reaction | 30 |
| | | Anaphylactic shock | 8 |
| **Skin and subcutaneous tissue disorders** | 31 | Rash maculo-papular | 12 |
| | | Urticaria | 7 |
| | | Angioedema | 7 |
| | | Rash | 5 |
| **Gastrointestinal disorders** | 7 | Abdominal pain | 1 |
| | | Oedema mouth | 1 |
| | | Nausea | 1 |
| | | Diarrhoea | 1 |

| | | | |
|---|---|---|---|
| **General disorders and administration site conditions** | 4 | Face oedema | 2 |
| | | Chest discomfort | 2 |
| **Hepatobiliary disorders** | 3 | Hepatitis cholestatic | 1 |
| | | Liver injury | 1 |
| | | Jaundice | 1 |

*Viscum album* L.

| **System Organ Class (SOC)** | **Frequency (n)** | **Preferred term (PT)** | **Frequency (n)** |
|---|---|---|---|
| **Skin and subcutaneous tissue disorders** | 29 | Urticaria | 12 |
| | | Pruritus | 9 |
| | | Angioedema | 4 |
| | | Erythema | 4 |
| **General disorders and administration site conditions** | 25 | Pyrexia | 10 |
| | | Chills | 7 |
| | | Injection site pain | 3 |
| | | Fatigue | 3 |
| **Immune system disorders** | 25 | Anaphylactic reaction | 12 |
| | | Hypersensitivity | 6 |
| | | Anaphylactic shock | 4 |
| | | Type I hypersensitivity | 1 |
| **Respiratory, thoracic and mediastinal disorders** | 17 | Dyspnoea | 5 |
| | | Bronchospasm | 3 |
| | | Respiratory distress | 3 |
| | | Pulmonary embolism | 1 |
| **Gastrointestinal disorders** | 10 | Vomiting | 3 |
| | | Nausea | 3 |
| | | Swollen tongue | 2 |
| | | Abdominal pain | 2 |

*Silybum marianum* (L.) Gaertn.

| **System Organ Class (SOC)** | **Frequency (n)** | **Preferred term (PT)** | **Frequency (n)** |
|---|---|---|---|
| **Gastrointestinal disorders** | 25 | Nausea | 10 |
| | | Diarrhoea | 7 |
| | | Abdominal pain upper | 5 |
| | | Vomiting | 3 |
| **Nervous system disorders** | 18 | Dizziness | 8 |
| | | Headache | 3 |
| | | Transient ischaemic attack | 1 |
| | | Somnolence | 1 |
| **Respiratory, thoracic and mediastinal disorders** | 17 | Dyspnoea | 8 |
| | | Cough | 2 |
| | | Throat tightness | 2 |
| | | Respiratory tract inflammation | 1 |

| | | | |
|---|---|---|---|
| **General disorders and administration site conditions** | 17 | Pyrexia | 3 |
| | | Malaise | 2 |
| | | Feeling hot | 2 |
| | | Death | 1 |
| **Skin and subcutaneous tissue disorders** | 16 | Pruritus | 5 |
| | | Hyperhidrosis | 5 |
| | | Rash | 3 |
| | | Angioedema | 3 |

*Astragalus membranaceus* Moench

| System Organ Class (SOC) | Frequency (n) | Preferred term (PT) | Frequency (n) |
|---|---|---|---|
| **General disorders and administration site conditions** | 18 | Chest pain | 9 |
| | | Chills | 7 |
| | | Pyrexia | 4 |
| | | Chest discomfort | 2 |
| **Respiratory, thoracic and mediastinal disorders** | 14 | Dyspnoea | 14 |
| **Cardiac disorders** | 5 | Palpitations | 5 |
| **Immune system disorders** | 4 | Anaphylactoid reaction | 2 |
| | | Anaphylactic reaction | 1 |
| | | Anaphylactic shock | 1 |
| **Psychiatric disorders** | 2 | Tic | 2 |
| | | Restlessness | 1 |

*Curcuma longa* L.

| System Organ Class (SOC) | Frequency (n) | Preferred term (PT) | Frequency (n) |
|---|---|---|---|
| **Gastrointestinal disorders** | 7 | Nausea | 3 |
| | | Diarrhoea | 3 |
| | | Abdominal pain | 2 |
| | | Abdominal distension | 1 |
| **General disorders and administration site conditions** | 5 | Pyrexia | 2 |
| | | Asthenia | 1 |
| | | Gait disturbance | 1 |
| | | Chest discomfort | 1 |
| **Hepatobiliary disorders** | 5 | Liver injury | 1 |
| | | Acute hepatic failure | 1 |
| | | Subacute hepatic failure | 1 |
| | | Hepatitis cholestatic | 1 |
| **Nervous system disorders** | 4 | Headache | 2 |
| | | Paraesthesia | 1 |
| | | Clonus | 1 |
| | | Dizziness | 1 |

| | | | |
|---|---|---|---|
| **Skin and subcutaneous tissue disorders** | 4 | Alopecia | 2 |
| | | Nail disorder | 1 |
| | | Pruritus | 1 |
| | | Erythema | 1 |

*Salvia officinalis* L.

| System Organ Class (SOC) | Frequency (n) | Preferred term (PT) | Frequency (n) |
|---|---|---|---|
| **Skin and subcutaneous tissue disorders** | 17 | Pruritus | 4 |
| | | Erythema | 4 |
| | | Alopecia | 3 |
| | | Eczema | 2 |
| **Gastrointestinal disorders** | 14 | Nausea | 3 |
| | | Oesophageal pain | 3 |
| | | Abdominal discomfort | 2 |
| | | Tongue movement disturbance | 1 |
| **Nervous system disorders** | 12 | Dizziness | 2 |
| | | Loss of consciousness | 2 |
| | | Hypoaesthesia | 1 |
| | | Seizure | 1 |
| **General disorders and administration site conditions** | 11 | Condition aggravated | 2 |
| | | Therapeutic response unexpected | 2 |
| | | Pain | 1 |
| | | Crying | 1 |
| **Psychiatric disorders** | 8 | Drug abuse | 2 |
| | | Poor quality sleep | 2 |
| | | Nightmare | 2 |
| | | Restlessness | 1 |

*Panax quinquefolius* L.

| System Organ Class (SOC) | Frequency (n) | Preferred term (PT) | Frequency (n) |
|---|---|---|---|
| **Skin and subcutaneous tissue disorders** | 14 | Urticaria | 8 |
| | | Rash pruritic | 2 |
| | | Pruritus | 2 |
| | | Erythema | 1 |
| **Respiratory, thoracic and mediastinal disorders** | 13 | Dyspnoea | 3 |
| | | Respiratory acidosis | 2 |
| | | Cough | 2 |
| | | Throat tightness | 2 |
| **Gastrointestinal disorders** | 12 | Abdominal pain | 3 |
| | | Nausea | 3 |
| | | Diarrhoea | 2 |
| | | Vomiting | 2 |

| | | | |
|---|---|---|---|
| **General disorders and administration site conditions** | 11 | Chest pain | 4 |
| | | Condition aggravated | 2 |
| | | Generalised oedema | 1 |
| | | Drug interaction | 1 |
| **Investigations** | 10 | Heart rate increased | 3 |
| | | Electrocardiogram | 3 |
| | | Blood pressure increased | 2 |
| | | Blood test | 2 |

*Camellia sinensis* (L.) Kuntze

| System Organ Class (SOC) | Frequency (n) | Preferred term (PT) | Frequency (n) |
|---|---|---|---|
| **Hepatobiliary disorders** | 13 | Hepatitis | 3 |
| | | Hepatitis toxic | 3 |
| | | Hepatitis acute | 3 |
| | | Jaundice | 2 |
| **Gastrointestinal disorders** | 4 | Haemorrhoidal haemorrhage | 1 |
| | | Constipation | 1 |
| | | Haemorrhoids | 1 |
| | | Dry mouth | 1 |
| **Nervous system disorders** | 3 | Headache | 2 |
| | | Amnesia | 1 |
| | | Dizziness | 1 |
| **Vascular disorders** | 3 | Hypertension | 2 |
| | | Phlebitis superficial | 1 |
| **Investigations** | 3 | Transaminases increased | 1 |
| | | Myoglobin blood present | 1 |
| | | Blood creatine phosphokinase increased | 1 |
| | | Liver function test abnormal | 1 |

*Echinacea purpurea* (L.) Moench

| System Organ Class (SOC) | Frequency (n) | Preferred term (PT) | Frequency (n) |
|---|---|---|---|
| **General disorders and administration site conditions** | 7 | Condition aggravated | 2 |
| | | Oedema peripheral | 1 |
| | | Therapeutic product ineffective | 1 |
| | | Pyrexia | 1 |
| **Skin and subcutaneous tissue disorders** | 6 | Rash | 3 |
| | | Rash pruritic | 1 |
| | | Urticaria | 1 |
| | | Stevens-Johnson syndrome | 1 |
| **Nervous system disorders** | 5 | Polyneuropathy | 1 |
| | | Somnolence | 1 |
| | | Seizure | 1 |
| | | Idiopathic intracranial hypertension | 1 |

| | | | |
|---|---|---|---|
| **Gastrointestinal disorders** | 5 | Nausea | 2 |
| | | Diarrhoea | 2 |
| | | Oral mucosal blistering | 1 |
| | | Tongue blistering | 1 |
| **Hepatobiliary disorders** | 4 | Hypertransaminasaemia | 1 |
| | | Liver injury | 1 |
| | | Jaundice | 1 |
| | | Hepatitis | 1 |

*Withania somnifera* (L.) Dunal

| System Organ Class (SOC) | Frequency (n) | Preferred term (PT) | Frequency (n) |
|---|---|---|---|
| **Gastrointestinal disorders** | 7 | Vomiting | 2 |
| | | Vomiting projectile | 2 |
| | | Diarrhoea | 2 |
| | | Nausea | 1 |
| **Psychiatric disorders** | 5 | Agitation | 2 |
| | | Suicidal ideation | 1 |
| | | Mood altered | 1 |
| | | Irritability | 1 |
| **General disorders and administration site conditions** | 4 | Pyrexia | 1 |
| | | Malaise | 1 |
| | | Chest pain | 1 |
| | | Crying | 1 |
| **Cardiac disorders** | 3 | Tachycardia | 2 |
| | | Palpitations | 2 |
| **Investigations** | 3 | White blood cell count increased | 1 |
| | | Eosinophil count increased | 1 |
| | | C-reactive protein increased | 1 |
| | | Aspartate aminotransferase increased | 1 |

*Aloe vera (L.)* Burm.f.

| System Organ Class (SOC) | Frequency (n) | Preferred term (PT) | Frequency (n) |
|---|---|---|---|
| **Hepatobiliary disorders** | 5 | Hepatotoxicity | 2 |
| | | Jaundice | 1 |
| | | Hypertransaminasaemia | 1 |
| | | Hepatitis toxic | 1 |
| **Skin and subcutaneous tissue disorders** | 3 | Dermatitis | 2 |
| | | Rash | 1 |
| | | Pustular psoriasis | 1 |
| **Renal and urinary disorders** | 2 | Acute kidney injury | 2 |

| | | | |
|---|---|---|---|
| **Musculoskeletal and connective tissue disorders** | 2 | Arthritis reactive | 1 |
| | | Arthralgia | 1 |
| **General disorders and administration site conditions** | 2 | Application site rash | 1 |
| | | Pain | 1 |
| | | Asthenia | 1 |

*Rhodiola rosea* L.

| System Organ Class (SOC) | Frequency (n) | Preferred term (PT) | Frequency (n) |
|---|---|---|---|
| **Nervous system disorders** | 6 | Headache | 3 |
| | | Dizziness | 2 |
| | | Mental impairment | 1 |
| | | Migraine | 1 |
| **Psychiatric disorders** | 4 | Agitation | 2 |
| | | Anxiety | 2 |
| | | Insomnia | 1 |
| | | Binge eating | 1 |
| **Investigations** | 2 | Alanine aminotransferase increased | 1 |
| | | Blood prolactin increased | 1 |
| | | Aspartate aminotransferase increased | 1 |
| **Cardiac disorders** | 2 | Ventricular tachycardia | 1 |
| | | Cardio-respiratory arrest | 1 |
| **Immune system disorders** | 1 | Anaphylactic reaction | 1 |

**Table S6**. Overview of the reported multi-ingredient natural products involved in an ICSR as sole suspect.

| Natural Product | Number of Reports (n) |
|---|---|
| *Salvia miltiorrhiza* Bunge | 18623 |
| *Glycyrrhiza glabra* L. | 1853 |
| *Echinacea purpurea* (L.) Moench | 1536 |
| *Zingiber officinale* Roscoe | 1378 |
| *Panax ginseng* C.A.Meyer | 1253 |
| *Achyranthes bidentata* Blume | 988 |
| *Silybum marianum* (L.) Gaertn. | 980 |
| *Andrographis paniculata* (Burm.f.) Wall. ex Nees | 836 |
| *Terminalia chebula* Retz. | 528 |
| *Allium sativum* L. | 504 |
| *Echinacea angustifolia* | 490 |
| *Aloe vera(L.)* Burm.f. | 371 |
| *Viscum album* L. | 354 |
| *Eleutherococcus senticosus* (Rupr. & Maxim.) Maxim. | 294 |
| *Curcuma longa* L. | 261 |
| *Camellia sinensis* (L.) Kuntze | 231 |
| *Withania somnifera* (L.) Dunal | 193 |
| *Salvia officinalis* L. | 175 |
| *Azadirachta indica* A. Juss | 162 |
| *Pelargonium sidoides* DC. | 159 |
| *Berberis vulgaris* L. | 137 |
| *Schisandra chinensis* (Turcz.) Baill. | 108 |
| *Rhodiola rosea* L. | 79 |
| *Astragalus membranaceus* Moench | 64 |
| *Phyllanthus emblica* L | 52 |
| *Bacopa monnieri* (L.) Wettst. | 50 |
| Propolis from *Apis mellifera* | 41 |
| *Panax notoginseng (*Burkill) F.H. Chen | 26 |
| *Ganoderma lucidum* (Curtis) P. Karst | 25 |
| *Momordica charantia* L. | 22 |
| *Carica papaya* L. | 20 |
| *Hericium erinaceus* (Bull.) Pers. | 15 |
| *Lycium barbarum* L. | 12 |
| *Ophiocordyceps sinensis* (Berk.) Sacc. | 12 |
| *Morinda citrifolia L* | 9 |
| *Panax quinquefolius* L | 9 |
| *Lentinula edodes* (Berk.) Pegl. | 8 |
| *Grifola frondosa (*Dicks.) Gray | 3 |
| *Uncaria tomentosa* (Willd. ex Schult.) DC. | 3 |
| *Aronia melanocarpa* (Michx.) Elliott | 2 |
| *Artemisia annua* L. | 2 |
| *Pleurotus ostreatus* (Jacq.Fr.) P. Kumm. | 1 |

**Table S7.** Overview of the top 15 most reported multi-ingredient natural products with the SOCs and their respective PTs.

*Salvia miltiorrhiza* Bunge

| System Organ Class (SOC) | Frequency (n) | Preferred term (PT) | Frequency (n) |
|---|---|---|---|
| **Gastrointestinal disorders** | 3789 | Nausea | 2195 |
| | | Vomiting | 1079 |
| | | Abdominal pain | 267 |
| | | Abdominal discomfort | 248 |
| **General disorders and administration site conditions** | 5647 | Chest pain | 2686 |
| | | Chills | 1814 |
| | | Pyrexia | 818 |
| | | Pain | 329 |
| **Nervous system disorders** | 3942 | Dizziness | 2071 |
| | | Headache | 1240 |
| | | Hypoaesthesia | 327 |
| | | Head discomfort | 286 |
| **Skin and subcutaneous tissue disorders** | 5043 | Pruritus | 2528 |
| | | Rash | 1609 |
| | | Erythema | 476 |
| | | Hyperhidrosis | 430 |
| **Vascular disorders** | 2870 | Phlebitis | 1471 |
| | | Flushing | 1156 |
| | | Pallor | 138 |
| | | Phlebitis superficial | 105 |

*Echinacea purpurea* (L.) Moench

| System Organ Class (SOC) | Frequency (n) | Preferred term (PT) | Frequency (n) |
|---|---|---|---|
| **Skin and subcutaneous tissue disorders** | 591 | Pruritus | 212 |
| | | Rash | 149 |
| | | Urticaria | 145 |
| | | Erythema | 85 |
| **Nervous system disorders** | 550 | Ageusia | 204 |
| | | Dysgeusia | 193 |
| | | Dizziness | 87 |
| | | Taste disorder | 66 |
| **Gastrointestinal disorders** | 484 | Nausea | 181 |
| | | Vomiting | 133 |
| | | Diarrhoea | 115 |
| | | Abdominal pain | 55 |
| **Immune system disorders** | 324 | Hypersensitivity | 164 |
| | | Anaphylactic reaction | 148 |
| | | Anaphylactic shock | 8 |
| | | Drug hypersensitivity | 4 |

| | | | |
|---|---|---|---|
| **General disorders and administration site conditions** | 144 | Malaise | 48 |
| | | Swelling face | 37 |
| | | Feeling hot | 31 |
| | | Fatigue | 28 |

*Glycyrrhiza glabra* L.

| System Organ Class (SOC) | Frequency (n) | Preferred term (PT) | Frequency (n) |
|---|---|---|---|
| **Gastrointestinal disorders** | 445 | Nausea | 144 |
| | | Vomiting | 104 |
| | | Diarrhoea | 102 |
| | | Abdominal pain upper | 95 |
| **General disorders and administration site conditions** | 386 | Drug ineffective | 227 |
| | | No adverse event | 78 |
| | | Malaise | 48 |
| | | Fatigue | 33 |
| **Skin and subcutaneous tissue disorders** | 370 | Rash | 130 |
| | | Pruritus | 129 |
| | | Urticaria | 71 |
| | | Erythema | 40 |
| **Product issues** | 257 | Product contamination physical | 82 |
| | | Product taste abnormal | 80 |
| | | Product quality issue | 75 |
| | | Product physical consistency issue | 20 |
| **Injury, poisoning and procedural complications** | 220 | Poor quality product administered | 128 |
| | | Off label use | 46 |
| | | Incorrect dose administered | 32 |
| | | Wrong technique in product usage process | 14 |

*Andrographis paniculata* (Burm.f.) Wall. ex Nees

| System Organ Class (SOC) | Frequency (n) | Preferred term (PT) | Frequency (n) |
|---|---|---|---|
| **Nervous system disorders** | 496 | Ageusia | 205 |
| | | Dysgeusia | 191 |
| | | Taste disorder | 67 |
| | | Headache | 33 |
| **Skin and subcutaneous tissue disorders** | 414 | Pruritus | 146 |
| | | Urticaria | 111 |
| | | Rash | 97 |
| | | Erythema | 60 |
| **Gastrointestinal disorders** | 303 | Nausea | 89 |
| | | Diarrhoea | 87 |
| | | Vomiting | 83 |
| | | Abdominal pain | 44 |

| | | | |
|---|---|---|---|
| **Immune system disorders** | 300 | Anaphylactic reaction | 153 |
| | | Hypersensitivity | 140 |
| | | Anaphylactic shock | 3 |
| | | Type IV hypersensitivity reaction | 2 |
| **General disorders and administration site conditions** | 111 | Malaise | 39 |
| | | Swelling face | 26 |
| | | Fatigue | 23 |
| | | Feeling hot | 23 |

*Zingiber officinale* Roscoe

| System Organ Class (SOC) | Frequency (n) | Preferred term (PT) | Frequency (n) |
|---|---|---|---|
| **Respiratory, thoracic and mediastinal disorders** | 331 | Interstitial lung disease | 246 |
| | | Lung disorder | 45 |
| | | Dyspnoea | 21 |
| | | Choking | 19 |
| **Hepatobiliary disorders** | 236 | Hepatic function abnormal | 97 |
| | | Drug-induced liver injury | 78 |
| | | Liver disorder | 40 |
| | | Jaundice | 21 |
| **Skin and subcutaneous tissue disorders** | 224 | Pruritus | 71 |
| | | Rash | 70 |
| | | Urticaria | 45 |
| | | Erythema | 19 |
| **Gastrointestinal disorders** | 154 | Dysphagia | 56 |
| | | Vomiting | 35 |
| | | Diarrhoea | 34 |
| | | Nausea | 29 |
| **General disorders and administration site conditions** | 92 | Pain | 24 |
| | | Swelling | 23 |
| | | Pyrexia | 23 |
| | | Malaise | 11 |

*Panax ginseng* C.A. Meyer

| System Organ Class (SOC) | Frequency (n) | Preferred term (PT) | Frequency (n) |
|---|---|---|---|
| **Gastrointestinal disorders** | 274 | Nausea | 107 |
| | | Diarrhoea | 67 |
| | | Vomiting | 60 |
| | | Abdominal pain | 40 |
| **Nervous system disorders** | 224 | Headache | 94 |
| | | Dizziness | 88 |
| | | Somnolence | 35 |
| | | Tremor | 7 |

| | | | |
|---|---|---|---|
| **Respiratory, thoracic and mediastinal disorders** | 207 | Interstitial lung disease | 161 |
| | | Lung disorder | 26 |
| | | Dyspnoea | 13 |
| | | Cough | 7 |
| **Hepatobiliary disorders** | 158 | Hepatic function abnormal | 75 |
| | | Drug-induced liver injury | 43 |
| | | Liver disorder | 27 |
| | | Jaundice | 13 |
| **General disorders and administration site conditions** | 80 | Fatigue | 25 |
| | | Pyrexia | 22 |
| | | Malaise | 18 |
| | | Drug ineffective | 15 |

*Achyranthes bidentata* Blume

| System Organ Class (SOC) | Frequency (n) | Preferred term (PT) | Frequency (n) |
|---|---|---|---|
| **Gastrointestinal disorders** | 448 | Dyspepsia | 306 |
| | | Nausea | 72 |
| | | Constipation | 39 |
| | | Gastritis | 31 |
| **Nervous system disorders** | 142 | Headache | 65 |
| | | Dizziness | 52 |
| | | Paraesthesia | 15 |
| | | Somnolence | 10 |
| **Infections and infestations** | 112 | Nasopharyngitis | 80 |
| | | Upper respiratory tract infection | 12 |
| | | Cystitis | 10 |
| | | Rhinitis | 5 |
| **Skin and subcutaneous tissue disorders** | 97 | Pruritus | 45 |
| | | Urticaria | 24 |
| | | Rash | 20 |
| | | Erythema | 4 |
| **General disorders and administration site conditions** | 89 | Face oedema | 30 |
| | | Oedema | 27 |
| | | Oedema peripheral | 18 |
| | | Chest pain | 14 |

*Silybum marianum* (L.) Gaertn

| System Organ Class (SOC) | Frequency (n) | Preferred term (PT) | Frequency (n) |
|---|---|---|---|
| **Gastrointestinal disorders** | 364 | Nausea | 94 |
| | | Diarrhoea | 78 |
| | | Vomiting | 68 |
| | | Nausea | 62 |

| | | | |
|---|---|---|---|
| **Skin and subcutaneous tissue disorders** | 187 | Pruritus | 73 |
| | | Rash | 62 |
| | | Urticaria | 26 |
| | | Erythema | 26 |
| **General disorders and administration site conditions** | 118 | Drug ineffective | 53 |
| | | Malaise | 27 |
| | | Fatigue | 21 |
| | | Discomfort | 17 |
| **Investigations** | 80 | Liver function test increased | 30 |
| | | Hepatic enzyme increased | 21 |
| | | Gamma-glutamyltransferase increased | 16 |
| | | Alanine aminotransferase increased | 13 |
| **Hepatobiliary disorders** | 62 | Jaundice | 21 |
| | | Drug-induced liver injury | 15 |
| | | Liver disorder | 13 |
| | | Hepatitis | 13 |

*Terminalia chebula* Retz.

| System Organ Class (SOC) | Frequency (n) | Preferred term (PT) | Frequency (n) |
|---|---|---|---|
| **General disorders and administration site conditions** | 254 | Drug ineffective | 143 |
| | | No adverse event | 77 |
| | | Malaise | 18 |
| | | Therapeutic product effect decreased | 16 |
| **Product issues** | 227 | Product contamination physical | 81 |
| | | Product quality issue | 69 |
| | | Product taste abnormal | 61 |
| | | Product physical consistency issue | 16 |
| **Injury, poisoning and procedural complications** | 178 | Poor quality product administered | 124 |
| | | Off label use | 39 |
| | | Wrong technique in product usage process | 10 |
| | | Product use complaint | 5 |
| **Gastrointestinal disorders** | 82 | Abdominal pain upper | 24 |
| | | Diarrhoea | 22 |
| | | Nausea | 20 |
| | | Constipation | 16 |
| **Nervous system disorders** | 62 | Dizziness | 27 |
| | | Paraesthesia | 17 |
| | | Headache | 13 |
| | | Head discomfort | 5 |

*Viscum album* L.

| System Organ Class (SOC) | Frequency (n) | Preferred term (PT) | Frequency (n) |
|---|---|---|---|
| **General disorders and administration site conditions** | 298 | Pyrexia | 143 |
| | | Local reaction | 77 |
| | | Chills | 18 |
| | | Pain | 16 |
| **Gastrointestinal disorders** | 63 | Abdominal discomfort | 24 |
| | | Nausea | 14 |
| | | Diarrhoea | 13 |
| | | Vomiting | 12 |
| **Skin and subcutaneous tissue disorders** | 42 | Pruritus | 16 |
| | | Urticaria | 12 |
| | | Rash | 10 |
| | | Blister | 4 |
| **Nervous system disorders** | 28 | Dizziness | 10 |
| | | Burning sensation | 7 |
| | | Headache | 5 |
| | | Seizure | 3 |
| **Respiratory, thoracic and mediastinal disorders** | 24 | Dyspnoea | 12 |
| | | Epistaxis | 4 |
| | | Cough | 4 |
| | | Nasal congestion | 2 |

*Echinacea angustifolia*

| System Organ Class (SOC) | Frequency (n) | Preferred term (PT) | Frequency (n) |
|---|---|---|---|
| **Gastrointestinal disorders** | 152 | Nausea | 77 |
| | | Vomiting | 45 |
| | | Diarrhoea | 18 |
| | | Dry mouth | 12 |
| **Skin and subcutaneous tissue disorders** | 115 | Pruritus | 44 |
| | | Rash | 26 |
| | | Urticaria | 23 |
| | | Erythema | 22 |
| **Nervous system disorders** | 84 | Dizziness | 46 |
| | | Headache | 21 |
| | | Somnolence | 5 |
| | | Seizure | 4 |
| **General disorders and administration site conditions** | 67 | Pain | 19 |
| | | Condition aggravated | 12 |
| | | Application site pain | 9 |
| | | Malaise | 9 |
| **Musculoskeletal and connective tissue disorders** | 27 | Osteoarthritis | 11 |
| | | Arthralgia | 8 |
| | | Pain in extremity | 4 |
| | | Myalgia | 4 |

*Allium sativum* L.

| System Organ Class (SOC) | Frequency (n) | Preferred term (PT) | Frequency (n) |
|---|---|---|---|
| **Gastrointestinal disorders** | 175 | Abdominal discomfort | 57 |
| | | Nausea | 52 |
| | | Dyspepsia | 37 |
| | | Diarrhoea | 29 |
| **Skin and subcutaneous tissue disorders** | 120 | Pruritus | 47 |
| | | Urticaria | 35 |
| | | Rash | 33 |
| | | Alopecia | 5 |
| **Nervous system disorders** | 41 | Dizziness | 22 |
| | | Headache | 12 |
| | | Somnolence | 4 |
| | | Dysgeusia | 3 |
| **General disorders and administration site conditions** | 31 | Malaise | 7 |
| | | Pain | 6 |
| | | Oedema peripheral | 6 |
| | | Face oedema | 6 |
| **Cardiac disorders** | 18 | Palpitations | 12 |
| | | Tachycardia | 2 |
| | | Cardiogenic shock | 1 |
| | | Atrial flutter | 1 |

*Aloe vera* (L.) Burm.f.

| System Organ Class (SOC) | Frequency (n) and % | Preferred term (PT) | Frequency (n) and % |
|---|---|---|---|
| **Skin and subcutaneous tissue disorders** | 122 | Pruritus | 44 |
| | | Rash | 36 |
| | | Erythema | 25 |
| | | Acne | 17 |
| **General disorders and administration site conditions** | 66 | Drug ineffective | 20 |
| | | Therapeutic response unexpected | 19 |
| | | Death | 16 |
| | | Fatigue | 11 |
| **Gastrointestinal disorders** | 47 | Nausea | 16 |
| | | Vomiting | 12 |
| | | Abdominal pain | 12 |
| | | Diarrhoea | 7 |
| **Injury, poisoning and procedural complications** | 24 | Off label use | 10 |
| | | Poor quality product administered | 5 |
| | | Fall | 5 |
| | | Hip fracture | 4 |

| | | | |
|---|---|---|---|
| **Nervous system disorders** | 23 | Dizziness | 7 |
| | | Headache | 6 |
| | | Loss of consciousness | 5 |
| | | Cerebrovascular accident | 5 |

*Eleutherococcus senticosus* (Rupr. & Maxim.) Maxim.

| System Organ Class (SOC) | Frequency (n) | Preferred term (PT) | Frequency (n) |
|---|---|---|---|
| **Gastrointestinal disorders** | 111 | Nausea | 37 |
| | | Vomiting | 31 |
| | | Diarrhoea | 27 |
| | | Dyspepsia | 16 |
| **Skin and subcutaneous tissue disorders** | 59 | Pruritus | 21 |
| | | Urticaria | 18 |
| | | Rash | 13 |
| | | Erythema | 7 |
| **Nervous system disorders** | 46 | Dizziness | 25 |
| | | Headache | 12 |
| | | Lethargy | 5 |
| | | Burning sensation | 4 |
| **General disorders and administration site conditions** | 37 | Face oedema | 9 |
| | | Fatigue | 7 |
| | | Pyrexia | 6 |
| | | Malaise | 5 |
| **Psychiatric disorders** | 17 | Insomnia | 9 |
| | | Agitation | 2 |
| | | Depression | 2 |
| | | Anxiety | 2 |

*Curcuma longa* L.

| System Organ Class (SOC) | Frequency (n) | Preferred term (PT) | Frequency (n) |
|---|---|---|---|
| **Gastrointestinal disorders** | 122 | Pruritus | 44 |
| | | Rash | 36 |
| | | Erythema | 25 |
| | | Acne | 17 |
| **Skin and subcutaneous tissue disorders** | 66 | Drug ineffective | 20 |
| | | Therapeutic response unexpected | 19 |
| | | Death | 16 |
| | | Fatigue | 11 |
| **Nervous system disorders** | 47 | Nausea | 16 |
| | | Vomiting | 12 |
| | | Abdominal pain | 12 |
| | | Diarrhoea | 7 |

| | | | |
|---|---|---|---|
| **General disorders and administration site conditions** | 24 | Off label use | 10 |
| | | Poor quality product administered | 5 |
| | | Fall | 5 |
| | | Hip fracture | 4 |
| **Investigations** | 23 | Dizziness | 7 |
| | | Headache | 6 |
| | | Loss of consciousness | 5 |
| | | Cerebrovascular accident | 5 |

**Table S8**. Search strategy applied to the scoping review in PubMed.

Search strategy of each specific species:

#1: ("Rhodiola"[Mesh] OR "rhodiola rosea"[tiab] OR "golden root"[tiab] OR "Withania"[Mesh] OR "withania somnifera"[tiab] OR Ashwagandha[tiab] OR "Eleutherococcus"[Mesh] OR "siberian ginseng"[tiab] OR "Acanthopanax senticosus"[tiab] OR "Eleutherococcus senticosus"[tiab] OR "Panax"[Mesh] OR "panax ginseng"[tiab] OR ginseng*[tiab] OR "Panax quinquefolius L."[tiab] OR "Panax pseudoginseng"[tiab] OR "Schisandra"[Mesh] OR "schisandra chinensis" [tiab] OR schisandra[tiab] OR "Astragalus propinquus"[Mesh] OR "astragalus membranaceus"[tiab] OR astragalus[tiab] OR "Curcuma"[Mesh] OR turmeric[tiab] OR "curcuma longa"[tiab] OR zingiberaceae [tiab] OR "Leuzea"[Mesh] OR "Rhaponticum carthamoides"[tiab] OR "Ocimum sanctum"[Mesh] OR "ocimum sanctum"[tiab] OR "Codonopsis"[Mesh] OR dangshen[tiab] OR "codonopsis pilosula"[tiab] OR "Tinospora"[Mesh] OR "Tinospora cordifolia"[tiab] OR "Bacopa"[Mesh] OR "bacopa monnieri"[tiab] OR "Phyllanthus emblica"[Mesh] OR "phyllanthus emblica"[tiab] OR "emblica officinalis"[tiab] OR "Glycyrrhiza"[Mesh] OR "glycyrrhiza glabra"[tiab] OR licorice[tiab] OR "asparagus racemosus"[tiab] OR "shatavari"[tiab] OR "Panax notoginseng"[Mesh] OR "panax notoginseng"[tiab] OR "Agaricus blazei" [Supplementary Concept] OR "agaricus blazei"[tiab] OR "Reishi"[Mesh] OR "lingzhi"[tiab] OR "ganoderma lucidum"[tiab] OR "Cordyceps"[Mesh] OR "cordyceps sinensis"[tiab] OR "cordyceps militaris"[tiab] OR "Trametes"[Mesh] OR "Trametes versicolor"[tiab] OR "turkey tail"[tiab] OR "Grifola"[Mesh] OR "grifola frondosa"[tiab] OR "Inonotus"[Mesh] OR "inonotus obliquus"[tiab] OR "chaga"[tiab] OR "Hericium"[Mesh] OR "hericium erinaceus"[tiab] OR "lion′s mane"[tiab] OR "Shiitake Mushrooms"[Mesh] OR "lentinula edodes"[tiab] OR shiitake[tiab] OR "Lepidium"[Mesh] OR "lepidium meyenii"[tiab] OR "lepidium peruvianum"[tiab] OR "Silybum marianum"[Mesh] OR "milk thistle"[tiab] OR "silybum marianum"[tiab] OR "Echinacea"[Mesh] OR "Echinacea purpurea"[tiab] OR "Garlic"[Mesh] OR garlic[tiab] OR "allium sativum"[tiab] OR "Zingiber officinale"[Mesh] OR ginger[tiab] OR "zingiber officinale"[tiab] OR "Cat's Claw"[Mesh] OR "uncaria tomentosa"[tiab] OR "cat′s claw"[tiab] OR "Aloe"[Mesh] OR "aloe vera"[tiab] OR "Andrographis paniculata"[Mesh] OR "chirata"[tiab] OR "andrographis paniculata"[tiab] OR "Camellia sinensis"[Mesh] OR "camellia sinensis"[tiab] OR "Azadirachta"[Mesh] OR "azadirachta indica"[tiab] OR "neem"[tiab] OR "Pleurotus"[Mesh] OR "pleurotus ostreatus"[tiab] OR "oyster mushroom"[tiab] OR "Terminalia"[Mesh] OR "Terminalia chebula"[tiab] OR "Haritaki"[tiab] OR "Pfaffia paniculata"[tiab] OR "Piper longum"[tiab] OR "dioscorea polystachya"[tiab] OR "Artemisia annua"[Mesh] OR "artemisia annua"[tiab] OR "sweet wormwood"[tiab] OR "Lithospermum"[Mesh] OR "lithospermum erythrorhizon"[tiab] OR "Lycium"[Mesh] OR "goji berry"[tiab] OR "lycium barbarum"[tiab] OR "Eriobotrya"[Mesh] OR "Eriobotrya japonica"[tiab] OR "loquat"[tiab] OR "Berberis"[Mesh] OR "barberry"[tiab] or "berberis vulgaris"[tiab] OR "Achyranthes"[Mesh] OR "achyranthes bidentata"[tiab] OR "Carica"[Mesh] OR "carica papaya"[tiab] OR "papaya"[tiab] OR "Morinda"[Mesh] OR "morinda citrifolia"[tiab] OR "Chromolaena"[Mesh] OR "chromolaena odorata"[tiab] OR "Spatholobus suberectus"[tiab] OR "Quillaja"[Mesh] OR "quillaja saponaria"[tiab] OR "dimocarpus longan"[tiab] OR "longan"[tiab] OR "Salvia officinalis"[Mesh] OR "salvia officinalis"[tiab] OR

"sage plants"[tiab] OR "Origanum"[Mesh] OR "oregano"[tiab] OR "origanum vulgare"[tiab] OR "Moringa oleifera"[Mesh] OR "moringa oleifera"[tiab] OR "Gynostemma"[Mesh] OR "gynostemma pentaphyllum*"[tiab] OR "Momordica charantia"[Mesh] OR "momordica charantia"[tiab] OR "bitter gourd"[tiab] OR "Orthosiphon"[Mesh] OR "orthosiphon stamineus"[tiab] OR "rhizoma gastrodiae"[tiab] OR "tian ma"[tiab] OR "Chenopodium quinoa"[Mesh] OR "chenopodium quinoa"[tiab] OR "quinoa*"[tiab] OR "Photinia"[Mesh] OR "aronia melanocarpa"[tiab] OR "black chokeberry"[tiab] OR "Antrodia"[Mesh] OR "antrodia cinnamomea"[tiab] OR "Pelargonium"[Mesh] OR "pelargonium sidoides"[tiab] OR "Viscum album"[Mesh] OR "viscum album"[tiab] OR "mistletoe"[tiab] OR "Propolis"[Mesh] OR "Propolis"[tiab] OR "Schizophyllum"[Mesh] OR "schizophyllum commune"[tiab])

#2: ("Drug-Related Side Effects and Adverse Reactions"[Mesh] OR "safe*"[tiab] OR "side effect*" [tiab] OR "adverse drug reaction*"[tiab] OR adr[tiab] OR "adverse reaction*"[tiab] OR "adverse drug reaction*"[tiab] OR "adverse event*"[tiab] OR "undesirable effect*" [tiab] OR "risk*"[tiab] OR "tolerability"[tiab] OR "toxic*"[tiab] OR "adverse effect*"[tiab])

#1 AND #2

Filter: case reports, clinical trials, randomized controlled trials and humans

Search strategy with general terms:

#1: ("Adaptation, Physiological"[Mesh] OR "Stress, Physiological"[Mesh] OR adaptogen*[tiab] OR "Adjuvants, Immunologic"[Mesh] OR "Immunologic Factors"[Mesh] OR immunomodulatory[tiab] OR immunomodulator*[tiab] OR immunomodulation[tiab] OR immunomodulate[tiab])

#2: ("Phytotherapy"[Mesh] OR "herbal products"[tiab] OR "plant extract*"[tiab] OR "herbal medicine*" [tiab] OR "Plants, Medicinal"[Mesh] OR "Herbal Medicine"[Mesh] OR "Plant Extracts"[Mesh] OR "Fungi"[Mesh] OR "Fungi"[tiab] OR "fungal"[tiab])

#3: ("Drug-Related Side Effects and Adverse Reactions"[Mesh] OR "safe*"[tiab] OR "side effect*" [tiab] OR "adverse drug reaction*"[tiab] OR adr[tiab] OR "adverse reaction*"[tiab] OR "adverse drug reaction*"[tiab] OR "adverse event*"[tiab] OR "undesirable effect*" [tiab] OR "risk*"[tiab] OR "tolerability"[tiab] OR "toxic*"[tiab] OR "adverse effect*"[tiab])

#1 AND #2 # AND #3

Filter: case reports, clinical trials, randomized controlled trials and humans.
